# Chimeric MHC class I– and II–restricted non-self epitopes broaden antitumor T cell reactions

**DOI:** 10.1084/jem.20250025

**Published:** 2025-12-05

**Authors:** Rongsheng Zhang, Rong Ma, Merrin M.L. Leong, Ian R. Watson, Kei Iida, Tomonori Yaguchi, Fumihiko Matsuda, Tasuku Honjo, Kenji Chamoto

**Affiliations:** 1 Department of Immunology and Genomic Medicine, Center for Cancer Immunotherapy and Immunobiology, Kyoto University Graduate School of Medicine, Kyoto, Japan; 2 Department of Human Genetics, McGill University, Montréal, Canada; 3 Rosalind and Morris Goodman Cancer Institute, McGill University, Montréal, Canada; 4 Department of Biochemistry, McGill University, Montréal, Canada; 5 Research Institute of the McGill University Health Centre, Montréal, Canada; 6 Faculty of Science and Engineering, Kindai University, Osaka, Japan; 7 Informatics Platform, Center for Cancer Immunotherapy and Immunobiology, Kyoto University Graduate School of Medicine, Kyoto, Japan; 8 Department of Immuno-Oncology PDT, Kyoto University Graduate School of Medicine, Kyoto, Japan; 9 Center for Genomic Medicine, Kyoto University Graduate School of Medicine, Kyoto, Japan

## Abstract

The mechanism by which one non-self antigen augments T cell immune responses to another remains unclear. We found that these expanded immune responses could derive from chimeric non-self peptides. These peptides, which we termed complete T cell antigens (CTAs), must be expressed intracellularly as single-chain chimeras containing both MHC class I– and II–restricted epitopes. CTAs, even unrelated to tumor antigens, when administered as live cell adjuvants or in cDNA-transfected muscle, increased T cell reactivity against tumor neoantigens. Mechanistically, CTA treatment altered dendritic cell phenotype in a CD4^+^ T cell–dependent manner, suppressing CD8^+^ T cell exhaustion and generating self-renewing CD8^+^ T cells in tumors. Cancers predicted to have long non-self peptides resulting from frameshift mutations, which frequently contain CTAs, were associated with a better prognosis or benefit from PD-1 blockade therapy in mouse models and cancer patients. These findings indicate that a subset of cancer cells expressing CTAs is sufficient to evoke overall antitumor immunity by broadening T cell responses to other neoantigens.

## Teaser

We identified peptide sequence principles that drive dendritic cell maturation and broaden tumor-reactive T cell clones, even with non-tumor sequences. These peptides must be expressed intracellularly in vivo as single chain containing MHC class I– and II–restricted non-self immunogenic epitopes.

## Graphical Abstract

**Figure:**
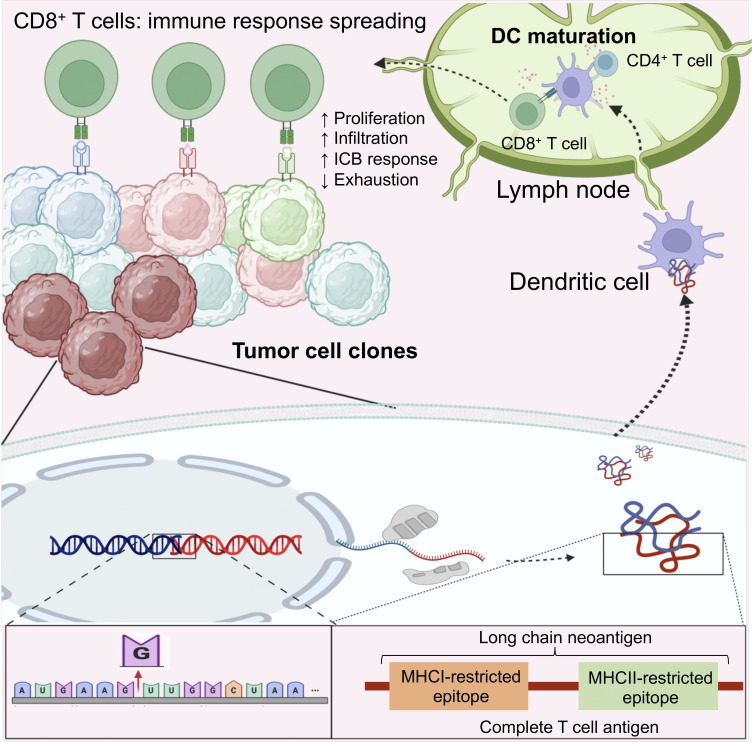

## Introduction

Immune checkpoint inhibition (ICI) can unleash antitumor immunity, and several such immunotherapies show impressive clinical activity against multiple cancer types. However, substantial proportion of patients remains unresponsive to treatment (Chen et al., 2024). To overcome ICI unresponsiveness, the factors that determine cancer immunogenicity must be investigated.

Both tumor cells and immune cells critically influence the antitumor immunity. MHC class II (MHC II)-restricted antigens presented by tumor cells play an important role in the tumor elimination (Alspach et al., 2019). A growing body of evidence has highlighted the importance of CD4^+^ T helper cells during immunotherapy. Increased CD4^+^ T cell percentages correlate with positive clinical outcomes after ICI treatment (Hatae et al., 2020; Kagamu et al., 2020). Tumor-specific CD4^+^ T cells provide essential support for tumor-specific CD8^+^ T cells. Immune reaction of CD4^+^ T cells modulate the tumor microenvironment (TME) through cytokine secretion or co-stimulation signals (Xiao et al., 2022; Schoenberger et al., 1998; Bennett et al., 1998) and help CD8^+^ T cell priming through modulating dendritic cells (DCs) (Ridge et al., 1998; Espinosa-Carrasco et al., 2024). CD8^+^ T cell infiltration of the TME is promoted by CD4^+^ T cells (Bos and Sherman, 2010). Furthermore, CD8^+^ T cells that receive help from CD4^+^ T cells have more potent effector functions and express fewer inhibitory immune checkpoint molecules than “helpless” CD8^+^ T cells (Ahrends et al., 2017; Espinosa-Carrasco et al., 2024).

The hallmark of CD8^+^ T cell responses in the TME or chronic viral infection is exhaustion (McLane et al., 2019), a dysfunctional state that occurs as an adaptation to chronic antigen exposure. Robust effector CD8^+^ T cells undergo a hierarchical loss of effector function during exhaustion, which leads to a state of hyporesponsiveness and eventual clonal deletion that are associated with impaired antitumor effects and cytotoxic activity (Bucks et al., 2009; Ahmadzadeh et al., 2009). In contrast, effector memory CD8^+^ tumor-infiltrating lymphocytes (TILs) possess stem-like features, including long-term persistence and spontaneous differentiation into terminally exhausted TILs. These effector memory T cells have proliferative capacity and respond to ICI by rapidly expanding, giving rise to effector T cells (Miller et al., 2019; LaFleur et al., 2019; Zhang et al., 2023).

Neoantigens are non-autologous proteins generated by non-synonymous mutations in the tumor cell genome (Zhang et al., 2021; Peng et al., 2019); they are not expressed in the normal tissues and exhibit strong immunogenicity. In addition to the transporters associated with antigen processing and T cell receptor (TCR) repertoire diversity (Lancaster et al., 2020; Battaglia, 2020; Wells et al., 2020; Jiang et al., 2019; Thaxton and Li, 2014; Cresswell et al., 2005), the affinity of mutant epitope for MHC class I (MHC I) or MHC II is one of the important determines whether these peptides could be tumor rejection antigens. A high tumor mutation burden (TMB) is associated with the accumulation of neoantigen-specific CD8^+^ TILs and better ICI responsiveness (Li et al., 2021; Fehlings et al., 2017; Gubin et al., 2014; Palmeri et al., 2022; Cristescu et al., 2022). As frameshift mutations generate a greater number of neoantigens than missense mutations, their frequency is reported to be associated with the efficacy of cancer immunotherapy (Sena et al., 2021; Chae et al., 2019; Maby et al., 2016). However, the fundamental mechanism by which neoantigens control the T cell responses across antigen specificities remains largely unknown.

This study aimed to investigate what type of mutations generate immunologically meaningful neoantigens that provoke the “chain reaction” of T cells to other neoantigens. We found that frameshift mutation-derived chimeric antigens consisting of MHC I– and MHC II–restricted non-self epitopes altered the DC phenotype, spreading CD8^+^ T cell responses to various different neoantigens. Our findings demonstrate the important role of the length, rather than the number, of neoantigen in tumor immunogenicity. This may provide new strategies for treating tumors that are unresponsive to ICI therapy.

## Results

### Complete T cell antigen suppresses tumor growth regardless of tumor antigen specificity

**Figure S1.**
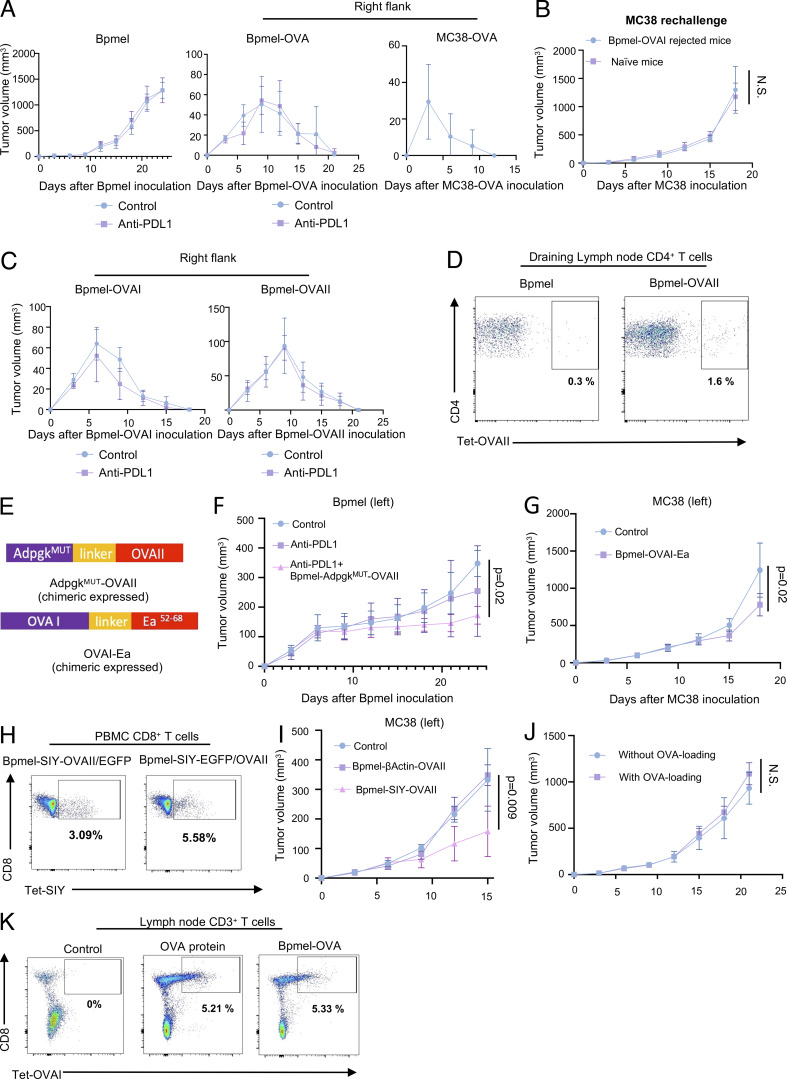
**Chimeric MHC I– and MHC II–restricted epitopes are essential for complete T cell antigen-induced antitumor effects. (A)** Tumor volumes in Bpmel (left; *n* = 5), Bpmel-OVAs (center; *n* = 5), or MC38-OVA (right; *n* = 10) mice treated with or without anti–PD-L1 therapy. **(B)** Tumor volumes in MC38-bearing mice injected 40 days after Bpmel-OVAI rejection; immune-naïve mice served as controls (*n* = 5). **(C) **Tumor volumes in Bpmel-OVAI (left) and Bpmel-OVAII (right) mice treated with or without anti–PD-L1 therapy (*n* = 5 per group). **(D)** OVAII-specific CD4^+^ T cells detected in DLN using OVAII-I-Ab tetramers 7 days after Bpmel-OVAII injection. **(E)** Schematic representation of AdpgkMUT-OVAII (upper panel) and OVAI-Ea (lower panel). **(F)** Bpmel tumor volume in mice treated with the Bpmel-AdpgkMUT-OVAII cell adjuvant on day 6 (*n* = 5 per group). **(G)** Tumor volumes in MC38-bearing mice treated with Bpmel-OVAI-Ea on day 6 (*n* = 5 per group). **(H)** SIY-specific CD8^+^ T cells detected in PBMCs using SIY-MHC tetramers 7 days after Bpmel-SIY-OVAII/EGFP or Bpmel-SIY-EGFP/OVAII injection. **(I)** Tumor volumes in MC38-bearing mice treated with Bpmel-β-actin-OVAII or Bpmel-SIY-OVAII on day 6 (*n* = 5). **(J)** Tumor growth in MC38-bearing mice receiving adoptive transfer of spleen cells with or without OVA loading on day 6 (*n* = 5 per group). **(K)** OVAI-specific CD8^+^ T cells detected in DLN cells using OVAI-MHC tetramers 7 days after Bpmel-OVA or OVA protein injection. Statistical analysis were determined using Student’s *t* test (F) or one-way ANOVA followed by Dunnett’s multiple comparison test (other panels). All data are representative of three similar experiments.

**Figure 1.**
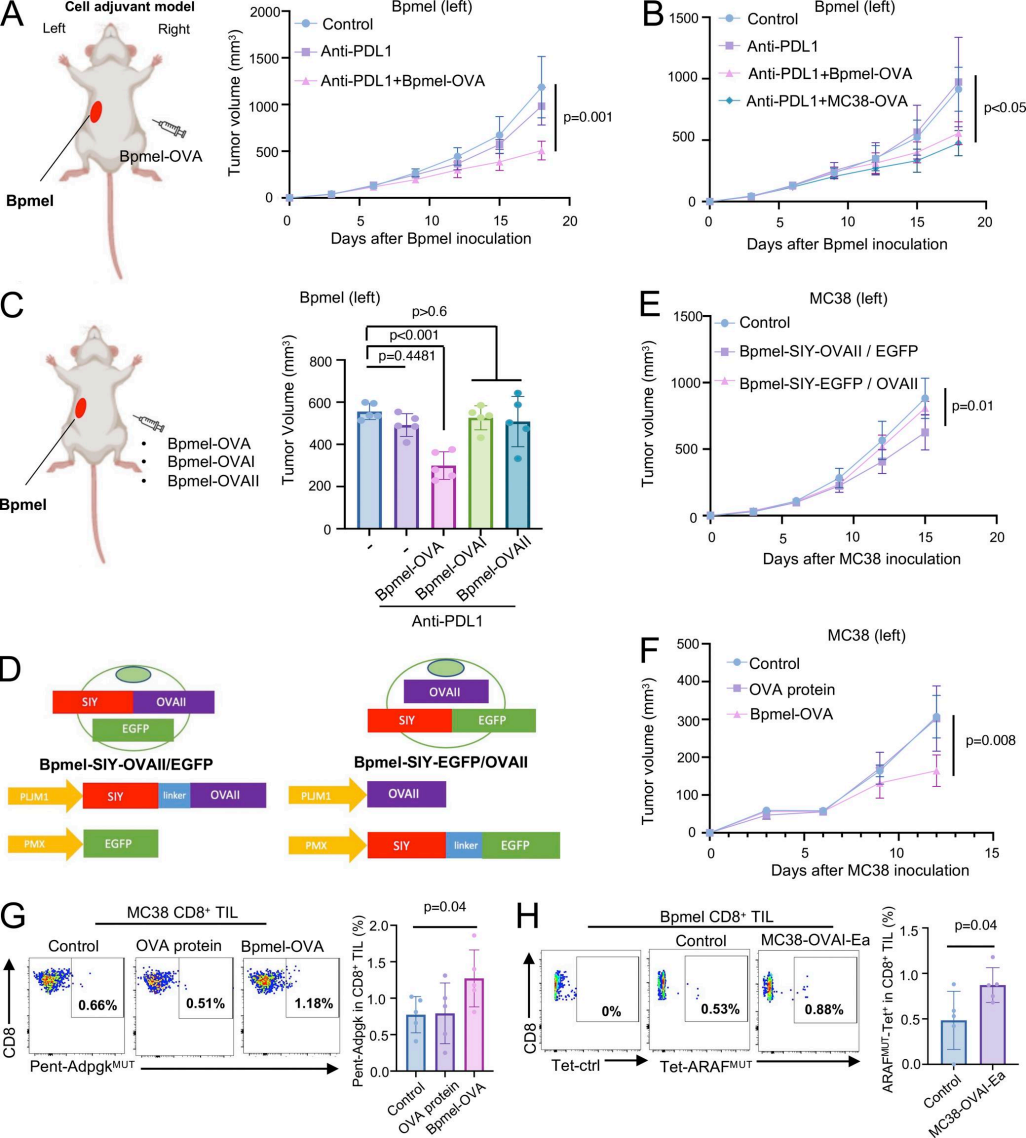
**Chimeric MHC I– and MHC II–restricted non-self peptides suppress tumor growth regardless of tumor antigen specificity. (A)** The cell adjuvant model schematic (left) and tumor volumes of the Bpmel (right; *n* = 5 per group). **(B)** Bpmel tumor volume after i.d. injection of Bpmel-OVA or MC38-OVA as cell adjuvants (*n* = 5 per group). **(C)** Bpmel tumor volume after injection of Bpmel-OVA, Bpmel-OVAI, or Bpmel-OVAII (*n* = 5 per group). **(D)** Schema of Bpmel-SIY-OVAII/EGFP and Bpmel-SIY-EGFP/OVAII. **(E)** MC38 tumor growth in C57BL/6-Tg (CAG-EGFP) mice following Bpmel-SIY-OVAII/EGFP or Bpmel-SIY-EGFP/OVAII injection (*n* = 5 per group). **(F)** Tumor volumes in MC38-bearing mice after i.d. injection of Bpmel-OVA cell adjuvant or OVA protein (200 μg/mouse) mixed with complete Freund’s adjuvant (*n* = 5 per group). **(G)** The frequency of Adpgk^MUT^-specific CD8^+^ T cells infiltrating MC38 tumors. **(H)** The frequency of ARAF^MUT^-specific CD8^+^ T cells among CD8^+^ T cells infiltrating Bpmel tumors after MC38-OVAI-Ea treatment (*n* = 5 per group). Student’s *t* test was used. P value was determined through one-way ANOVA followed by Dunnett’s multiple comparisons test (except H). All data are representative of two to three similar experiments.

To elucidate the mechanism by which immunogenic antigens induce antitumor immunity, we established a melanoma cell line, Bpmel, from gene-engineered mice (B6.Cg-Braf^tm1Mmcm^ Pten^tm1Hwu^ Tg[Tyr-cre/ERT2]13Bos/BosJ) (Nakamura et al., 2024), which was unresponsive to PD-1 blockade therapy (Fig. S1 A). We constructed Bpmel cells expressing the highly immunogenic OVA protein (Bpmel-OVA). When Bpmel-OVA cells were injected into the contralateral flank of Bpmel tumor–bearing mice as a “cell adjuvant,” Bpmel-OVA cells were naturally rejected (Fig. S1 A), and the primary Bpmel tumors in the opposite flank became responsive to PD-1 blockade therapy (Fig. 1 A). Injection of MC38-OVA, another highly immune-reactive tumor cell line, into Bpmel tumor–bearing mice yielded a similar effect (Fig. S1 A and Fig. 1 B). Rechallenge experiment demonstrated that MC38 and Bpmel did not share neoantigens (Fig. S1 B). These data indicate that the enhanced ICI sensitivity by the cell adjuvant treatment was due to the OVA protein. While OVA protein contains several immunogenic epitopes, two of them are widely used in immunology investigation: MHC I–binding OVA_257-264_ (OVAI) and MHC II–binding OVA_323-339_ (OVAII) (Lugade et al., 2005). We generated Bpmel strains expressing OVA I (Bpmel-OVAI) or OVA II (Bpmel-OVAII) to determine which epitope was responsible for the cell adjuvant effect (Tables S1 and S2). These cell lines exhibited high immunogenicity and were naturally rejected (Fig. S1 C). Although OVAII-specific CD4^+^ T cells were detected in draining LN (DLN) of Bpmel-OVAII tumor bearing mice (Fig. S1 D), only Bpmel-OVA but not Bpmel-OVAI or Bpmel-OVA-II exhibited an adjuvant effect, indicating that both MHC I and II epitopes were required for the cell adjuvant effect (Fig. 1 C). To rule out model antigen specificity, we generated other Bpmel transfectants: Bpmel-Adpgk^MUT^-OVAII (Adpgk^MUT^ as an MHC I–restricted epitope) (Hos et al., 2019) and Bpmel-OVAI-Ea (Ea ^52-68^ as an MHC II–restricted epitope) (Tables S1 and S2; and Fig. S1 E) (Iwamoto et al., 1996). Both cell adjuvants enhanced the antitumor activity (Fig. S1, F and G), indicating that immunity stimulated by non-self immunogenic MHC I and II peptides could enhance antitumor effects.

Next, we investigated whether the MHC I and II epitopes must be in the same peptide chain to confer the adjuvant effect. To this end, we constructed two cell lines: Bpmel-SIY-OVAII/EGFP, which expressed the MHC I–restricted SIY peptide and OVA II in the same peptide chain (Stone and Stern, 2006) (Table S2), and Bpmel-SIY-EGFP/OVAII, which expressed SIY and OVAII in different peptides (Fig. 1 D). We inoculated these cells into the C57BL/6-EGFP Tg (CAG-EGFP) mouse strain to exclude the immune reaction of the selection marker EGFP. Both cell lines generated SIY-tetramer^+^ CD8^+^ T cells at similar levels in the periphery (Fig. S1 H), but only Bpmel-SIY-OVAII/EGFP cell adjuvant exhibited an antitumor effect (Fig. 1 E). These data indicate that the MHC I and II epitopes must be chimeric in a single chain. Given that the presentation process of MHC II–restricted epitopes can be influenced by antigen length, we replaced the SIY epitope of SIY-OVAII with the same length of segment derived from murine self β-actin and constructed Bpmel-β-actin-OVAII (Table S2). Bpmel-β-actin-OVAII lost its antitumor effect compared with Bpmel-SIY-OVAII (Fig. S1 I), further confirming that MHC-I–restricted non-self epitope was required for enhancement of the antitumor effect.

When we compared the antitumor effects of OVA protein immunization with complete Freund’s adjuvant and the Bpmel-OVA cell adjuvant, only the Bpmel-OVA cell adjuvant showed antitumor activity in MC38-bearing mice (Fig. 1 F). Adoptive transfer of OVA-loaded spleen cells also failed to recapitulate the antitumor effect observed with cell adjuvants expressing OVA (Fig. S1 J) (Carbone and Bevan, 1990). MC38 neoantigen (Adpgk^MUT^)-specific CD8^+^ T cells markedly accumulated in tumor sites following treatment with the Bpmel-OVA cell adjuvant compared with OVA protein immunization, whereas OVA-specific immune responses were observed in both treatments (Fig. 1 G and Fig. S1 K). These data indicate that direct immunization with protein-form chimeric antigens is not sufficient for the enhancement of antitumor immunity, but the chimeric antigens need to be intracellularly expressed.

**Figure S2.**
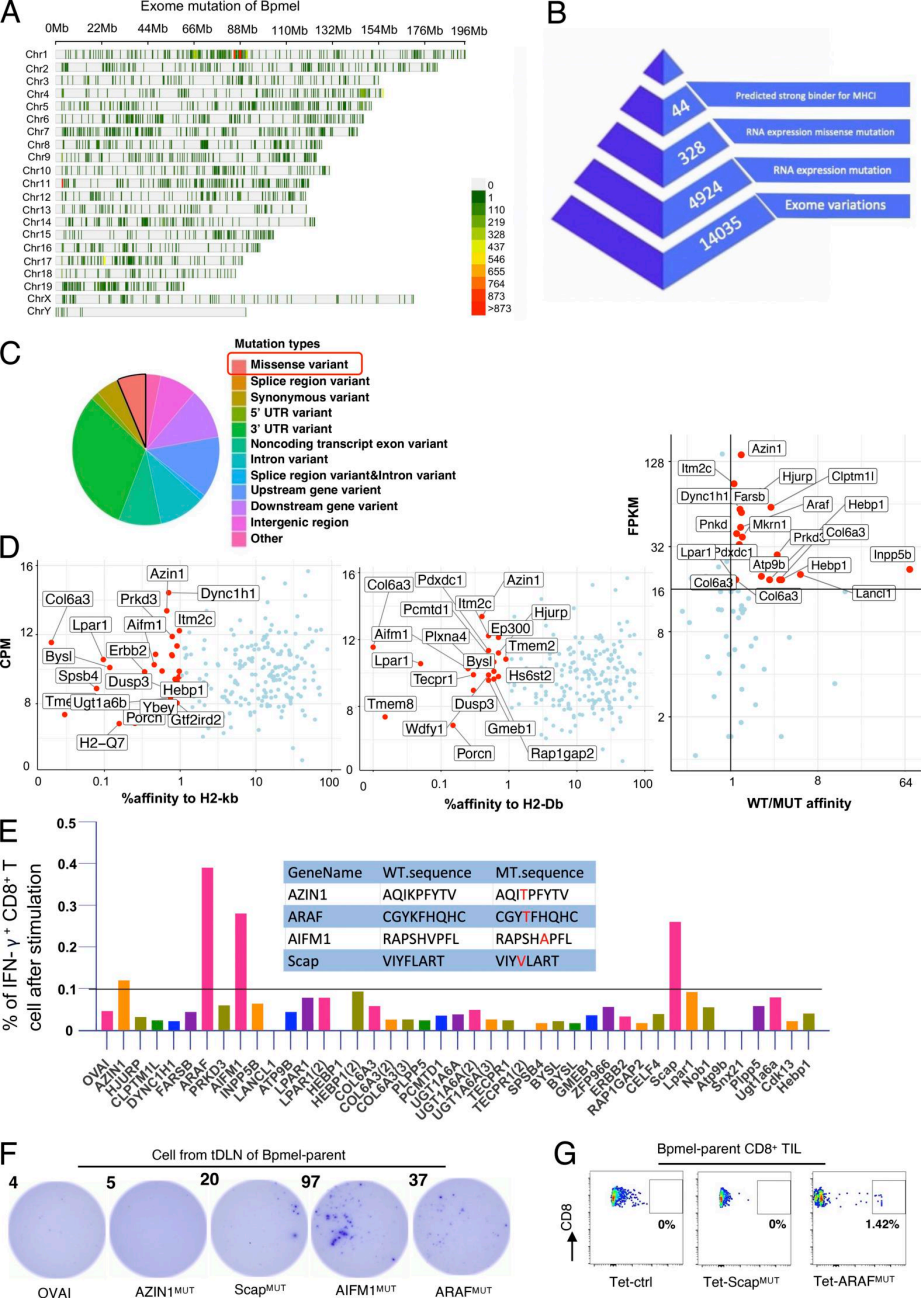
**Identification of neoantigens of Bpmel. (A)** Distribution of exome mutations at the chromosomal level in Bpmel. Colors indicate the number of mutations. This WES analysis identified 14,035 exomic mutations. **(B)** Workflow for the identification of Bpmel neoantigens. The numbers of peptide candidates screened at each step are shown. **(C)** Mutation types of Bpmel, as identified through WES analysis. At the RNA level, 328 missense variants were identified in 4,934 genes. **(D)** The 328 epitopes generated by missense mutation were screened based on their predicted affinities for H2-Kb and H2-Db. The top 1% of the affinity ranking was selected as high-affinity peptides (left and center). Candidates were further selected based on the ratio of affinity before and after mutation and on fragments per kb of transcript per million mapped reads (FPKM) (right). This screening identified 44 candidates (detailed sequences are listed in Table S3). **(E)** The 44 selected peptides were synthesized for the in vitro T cell screening assay. CD8^+^ T cells were isolated from the DLNs of Bpmel-bearing mice and stimulated with the indicated peptides (5 µg/ml) for 8 h. The percentage of IFN-γ–releasing CD8^+^ T cells was evaluated using IFN-γ–catching assays. Four WT and mutant peptide sequences that induced IFN-γ secretion are shown (center). **(F)** DLN single cells were stimulated with the indicated peptides (5 µg/ml) for 8 h. IFN-γ secretion was detected using the ELISpot assay. OVAI was used as a negative control. Dot numbers are shown. All four candidates except AZIN1^MUT^ promoted CD8^+^ T cell release of IFN-γ. **(G)** Control tetramers (Tet-ctrl), Scap^MUT^, and ARAF^MUT^ tetramers (the AIFM^MUT^ MHC tetramer could not be constructed) were used to stain CD8^+^ TILs. Here, 0.17–3.03% of ARAF^MUT^-specific CD8^+^ T cells were detected in the CD8^+^ TILs of all Bpmel-bearing mice (*n* = 5 per group). Representative images are shown.

We sought to identify the tumor neoantigen of Bpmel to further investigate whether the chimeric structure containing nonspecific MHC I and II epitopes enhanced tumor-specific T cell responses, even in unresponsive tumors. Whole-exome sequencing (WES), RNA sequencing (RNAseq), and affinity prediction for MHC I revealed 44 candidate missense-mutated peptides in Bpmel (Fig. S2, A–D; and Table S3). Further assays of the immune reaction and MHC tetramer among these 44 candidates demonstrated that the missense-mutated antigen ARAF^MUT^ (CGYTFHQHC) was the most immunoreactive (Fig. S2, E–G). The MC38-OVAI-Ea cell adjuvant treatment of Bpmel tumor–bearing mice increased the count of tumor-infiltrating ARAF^MUT^-specific CD8^+^ T cells in the tumor (Fig. 1 H).

These results indicated that the intracellular expression of non-self epitopes restricted by MHC I and MHC II that were chimeric in a single-peptide chain provided broad-spectrum tumor-specific immunity regardless of the tumor antigen specificity. We referred to antigens with such a structure as “complete T cell antigens" (CTAs).

### CTA suppresses CD8^+^ TIL exhaustion and promotes effector memory CD8^+^ T cell infiltration

**Figure S3.**
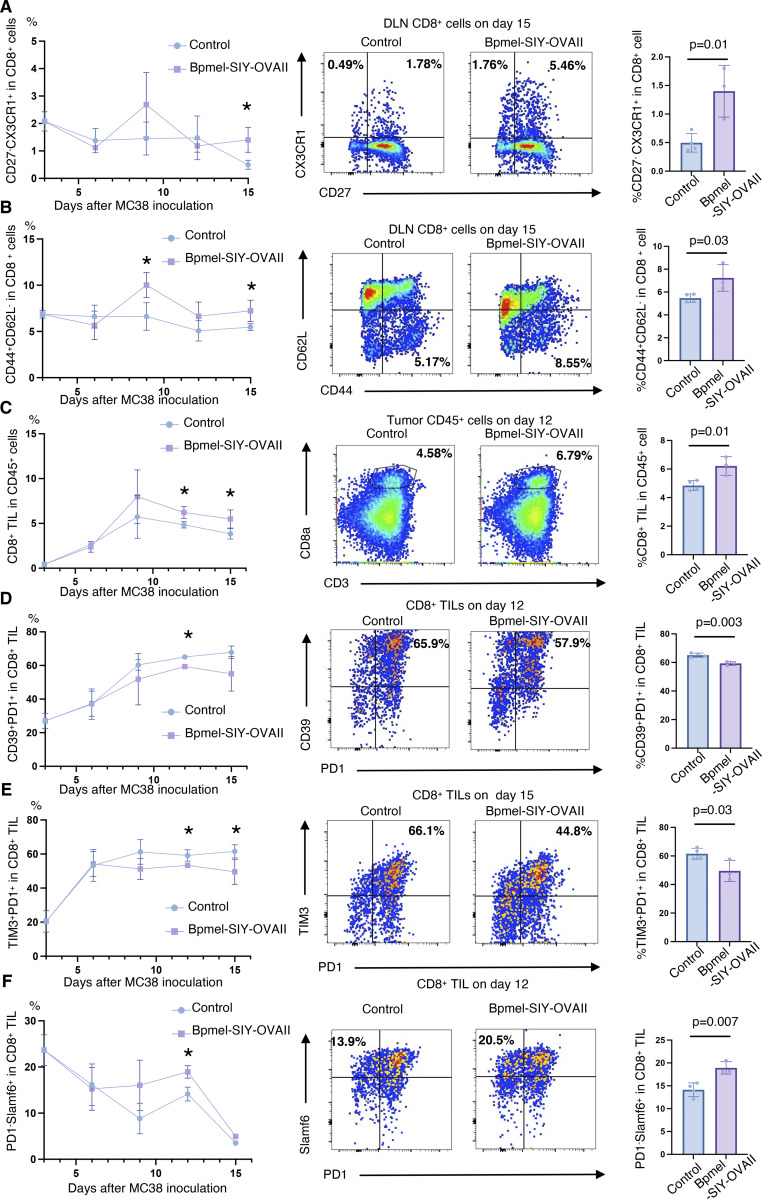
**Complete T cell antigen promotes the infiltration of self-replicable CD8** ^**+**^ **T cells and suppresses their exhaustion in the tumor. (A and B)** The frequency of CD8^+^ T cell fractions indicated by antibodies in the DLNs of MC38 tumor-bearing mice treated with or without Bpmel-SIY-OVAII cell adjuvant, as investigated every 3 days (left, *n* = 3 per group per time point). Representative images and their frequencies on day 15 are also shown (right). **(C–F)** The frequency of CD8^+^ T cell fractions defined by the indicated cell surface markers in tumors with or without Bpmel-SIY-OVAII cell adjuvant treatment, as investigated every 3 days (left, *n* = 3 per group per time point). Representative images and frequencies at the indicated time points are shown (right). Student’s *t* test was used for statistical analysis. *P < 0.05. All data are representative of three similar experiments.

Analysis of MC38 tumor-bearing mice treated with the Bpmel-SIY-OVAII cell adjuvant revealed a sustained increase in the proportion of CD27^−^ CX3CR1^+^ and CD62L^−^ CD44^+^ effector CD8^+^ T cells in the DLN as well as more significant infiltration of CD8^+^ T cells in the tumor site (Fig. S3, A–C) (Zwijnenburg et al., 2023; Yan et al., 2018; Gerlach et al., 2016). Terminally exhausted CD8^+^ T cells, marked by CD39^+^ PD1^+^ or TIM3^+^ PD1^+^, at the tumor site were significantly suppressed by cell adjuvant therapy (Fig. S3, D and E). In contrast, Slamf6^+^ PD1^−^, which represents a self-replicable CD8^+^ TIL population, was significantly increased (Fig. S3 F) (Miller et al., 2019). These results indicated that CTA treatment suppressed CD8^+^ TIL exhaustion and enhanced proliferative effector accumulation at the tumor site.

**Figure 2.**
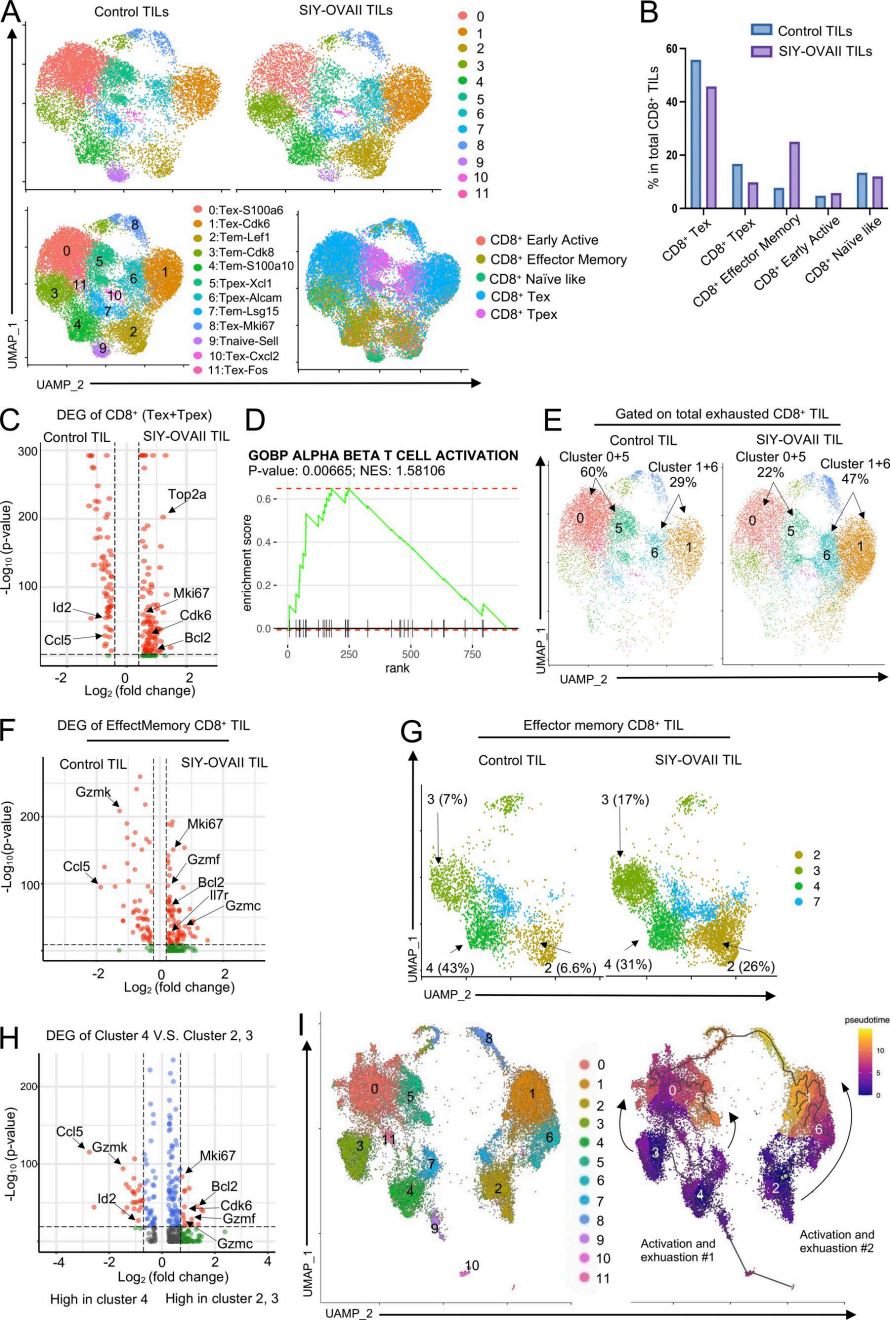
**Complete T cell antigen suppresses CD8** ^**+**^ **TIL exhaustion and promotes infiltration of effector memory CD8** ^**+**^ **T cells.** The MC38 tumor-bearing mice received Bpmel-SIY-OVAII cell therapy on day 6 after MC38 inoculation, and CD8^+^ TILs were subjected to scRNAseq on day 13. **(A)** In the upper panel, uniform manifold approximation and projection (UMAP) of CD8^+^ TILs treated with or without Bpmel-SIY-OVAII cell adjuvant (control TIL: *n* = 10,357 and SIY-OVAII TIL: *n* = 10,357). The name of each cluster and annotations of the cell types labelled with ProjecTILs were shown in the lower panel. **(B)** Percentages of each CD8^+^ TIL population based on the annotation by ProjecTILs. **(C)** A volcano plot showing differential expression of genes (DEGs) in total exhausted CD8^+^ TILs (Tex + Tpex) between the control and SIY-OVAII groups. **(D)** The GSEA enriched the pathway of “GOBP ALPHA BETA T CELL ACTIVATION” in CD8^+^ exhausted T cells between the control and SIY-OVAII TIL groups. **(E)** UMAP of total Tex and Tpex CD8^+^ TILs from the control and SIY-OVAII groups. **(F)** A volcano plot showing DEGs of CD8^+^ effector memory TILs between the control and SIY-OVAII groups. **(G)** UMAP of CD8^+^ effector memory TILs from the control or SIY-OVAII groups. **(H)** A volcano plot showing DEGs between clusters 4 vs. 2 plus 3. **(I)** UAMP dimension reduction was performed using Monocle3 with Seurat cluster labels (left), and pseudo-time values were calculated and plotted (right). GSEA, gene set enrichment analysis.

**Figure S4.**
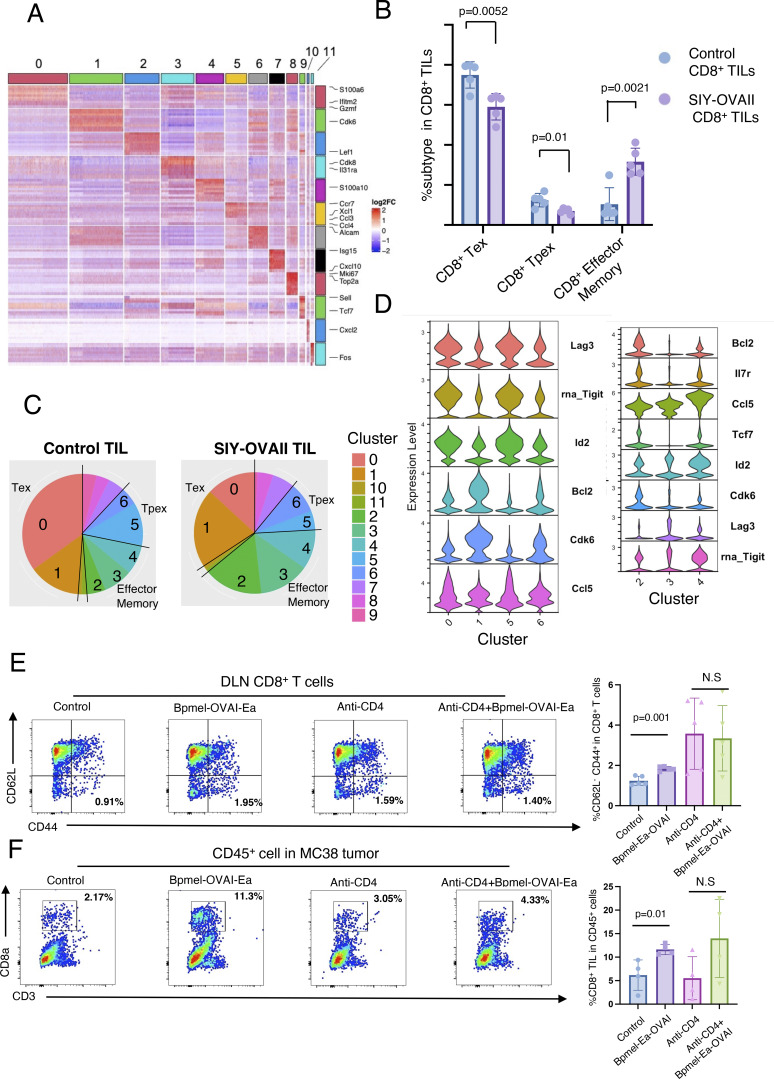
**Properties of each CD8** ^**+**^ **TIL cluster. (A)** A heatmap of DEGs among the 11 clusters shown in Fig. 2 A. **(B)** Subpopulation of CD8^+^ TILs from MC38 tumor-bearing mice with or without Bpmel-SIY-OVAII therapy (*n* = 5 per group). The analysis is based on flow cytometry; all data are representative of three similar experiments. (Tex, terminally exhausted T cell; Tpex, progenitor-exhausted T cells); statistical analysis was performed using *t* test. **(C)** The percentage of each cluster in the control and SIY-OVAII TILs shown in Fig. 2 E. **(D)** Violin plots of exhaustion-related markers and naïve/proliferation markers for the indicated clusters. **(E)** The frequency of effector CD8^+^ T cells (CD62L^−^ CD44^+^ population) in the DLN on day 21 (*n* = 5 per group). **(F)** The percentages of tumor-infiltrating CD8^+^ T cells in MC38 tumors on day 21 (*n* = 4 per group). Statistical analysis was performed using one-way ANOVA followed by Dunnett’s multiple comparison. All data are representative of three similar experiments.

We further performed single-cell RNAseq (scRNAseq) to analyze phenotypic changes in total CD8^+^ TILs of MC38 tumors treated with or without Bpmel-SIY-OVAII cell adjuvant therapy (hereafter referred to as SIY-OVAII TILs and control TILs, respectively). Unsupervised clustering analysis identified 11 distinct clusters, with no significant batch effects observed between the two conditions (Fig. 2 A, upper panel). Differentially expressed genes (DEGs) were identified for each cluster and annotated using their top marker genes (Fig. 2 A, lower left panel; and Fig. S4 A). To further characterize their functional states, CD8^+^ TILs were classified into naïve-like, early active, effector memory, precursor-exhausted (CD8^+^ Tpex), and exhausted (CD8^+^ Tex) subtypes using the ProjecTILs reference framework (Andreatta et al., 2021a) (Fig. 2 A, lower right panel). Based on this classification, we found a reduced frequency of Tex and Tpex CD8^+^ T cells and an increased proportion of effector memory CD8^+^ T cells in the SIY-OVAII group compared with the control group (Fig. 2 B); this result was further confirmed by flow cytometry analysis (Fig. S4 B).

To explore qualitative changes in the exhausted CD8^+^ T cell population following SIY-OVAII therapy, we compared gene expression profiles between the two groups. Tex and Tpex populations from SIY-OVAII TILs exhibited higher expression of *Mki67*, *Top2a*, *Cdk6*, and *Bcl2* compared with the control group, indicating enhanced proliferative capacity and increased resistance to apoptosis (Fig. 2 C). Gene set enrichment analysis of DEGs further supported this observation by revealing enrichment of T cell activation pathways in the SIY-OVAII group (Fig. 2 D). A closer examination showed that these phenotypic shifts were primarily driven by an increased proportion of clusters 1 and 6 (from 29% to 47%) and a corresponding reduction in clusters 0 and 5 (from 60% to 22%) (Fig. 2 E and Fig. S4 C). Notably, clusters 0 and 5 were enriched for exhaustion-related genes, such as *Lag3*, *Tigit*, and *Id2*, whereas clusters 1 and 6 predominantly expressed naïve or proliferative markers, including *Il7r*, *Bcl2*, and *Cdk6* (Fig. S4 D).

Within the effector memory population, SIY-OVAII TILs showed increased expression of proliferation-associated genes (*Il7r* and *Mki67*) and cytolytic effectors (*Gzmf* and *Gzmc*) compared with control TILs (Fig. 2 F). Interestingly, the proportions of clusters 2 and 3 were markedly elevated following SIY-OVAII therapy, while the proportion of cluster 4 decreased (Fig. 2 G). Clusters 2 and 3 were characterized by higher expression of proliferation markers (*Mki67* and *Cdk6*), anti-apoptotic genes (*Bcl2*), and backup granzymes (*Gzmf* and *Gzmc*), along with reduced expression of exhaustion markers such as *Ccl5*, *Id2*, and Tigit compared with cluster 4 (Fig. 2 H and Fig. S4 D) (Corria-Osorio et al., 2023). These results suggest that clusters 2 and 3 represent less exhausted, but more proliferative subsets than cluster 4.

Overall, CTA therapy may reduce the exhausted population and skew the phenotype toward a “younger” effector memory population with proliferative capacity. Trajectory analysis using the Monocle3 package revealed two possible transition paths (Fig. 2 I): from clusters 3 and 4 to clusters 0 and 5 (path #1) and from cluster 2 to clusters 1 and 6 (path #2). Therefore, SIY-OVAII therapy might favor enhancing the infiltration of “young” cluster 2, leading to the predominant clusters 1 and 6 in the exhausted SIY-OVAII TIL population (Fig. 2 E).

### CD4^+^ T cells are indispensable for CTA-induced antitumor effects

**Figure 3.**
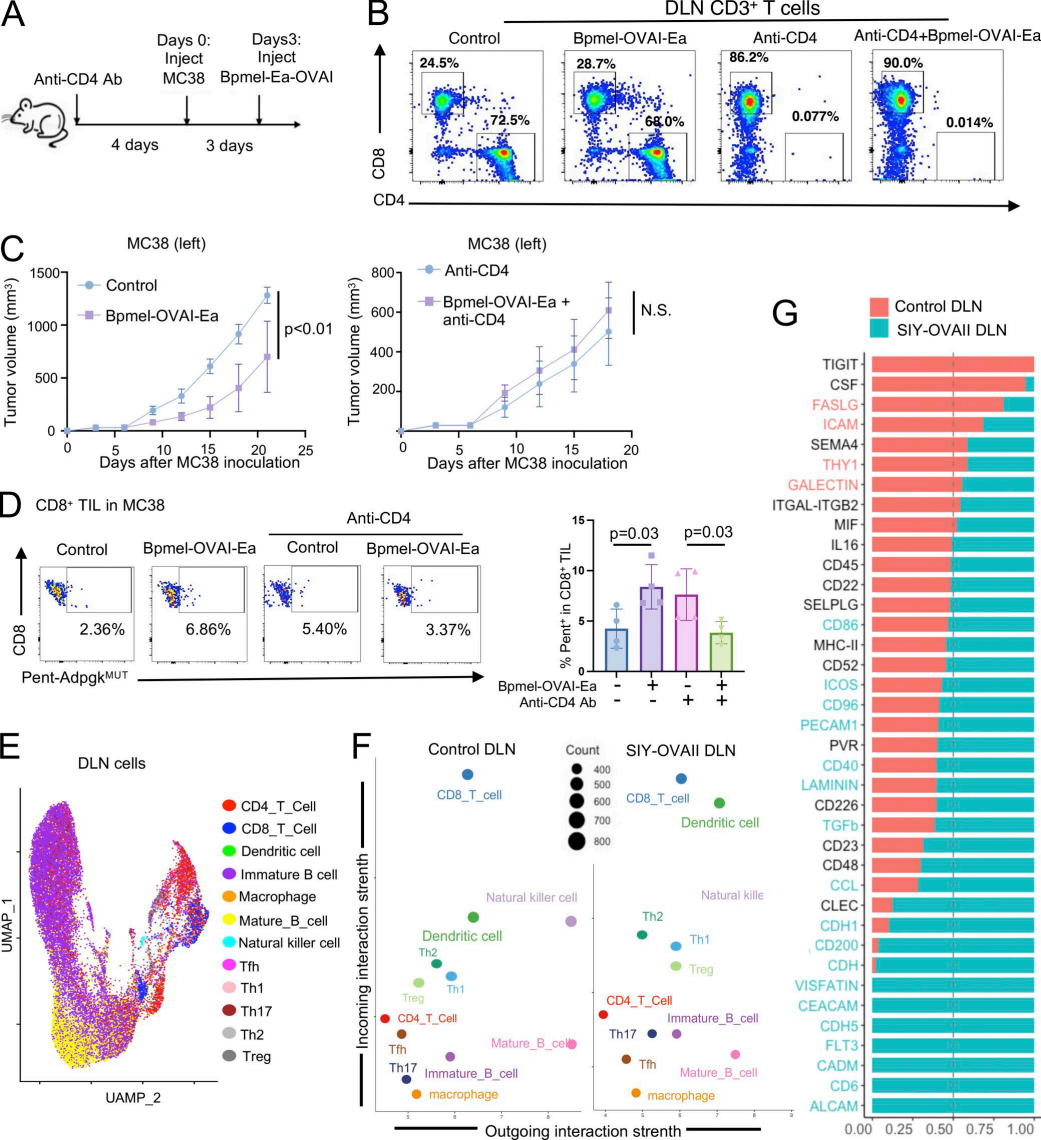
**Complete T cell antigen alters the** **DC** **phenotype of DLN in a CD4** ^**+**^ **T cell–dependent manner. (A)** Experimental schedule for CD4^+^ T cell depletion in the MC38 model. **(B)** Effects of CD4^+^ T cell depletion in the DLN cells on day 20. **(C)** MC38 tumor volumes following Bpmel-OVAI-Ea treatment with or without CD4^+^ T cell depletion (*n* = 5 per group). **(D)** Adpgk^MUT^-specific CD8 TIL infiltration (*n* = 4 per group). Student’s *t* test (C) and one-way ANOVA followed by Dunnett’s multiple comparison was used (D). **(E–G)** scRNAseq analysis of DLN cells, excluding naive T cells, from MC38-bearing mice treated with or without Bpmel-SIY-OVAII on day 6 (control: *n* = 10,952 and SIY-OVAII: *n* = 18,440 cells); (E) UMAP of the two group-mixture cells; (F) a scatter plot of incoming and outgoing interaction strengths for each population; (G) communication probabilities of the indicated signals in DC of the two groups. The relative information flow is the ratio of the communication probability between the experimental groups. Red and blue (Y axis) indicate significant enrichment in the control and SIY-OVAII DLNs, respectively; all data except E–G are representative of two to three similar experiments. UMAP, uniform manifold approximation and projection.

As MHC II–restricted epitopes were required for CTA-mediated antitumor effects, we hypothesized that CD4^+^ T cells were essential for these processes. To test this hypothesis, MC38 tumor-bearing mice were depleted of CD4^+^ T cells by injecting an anti-CD4 antibody followed by Bpmel-OVAI-Ea cell adjuvant therapy (Fig. 3, A and B). Although depletion of CD4^+^ T cells suppressed overall tumor volume, possibly through depletion of regulatory T cells (Tregs) (Kim et al., 2021), CD4 depletion abrogated antitumor activity of CTA (Fig. 3 C). CD4 depletion also abolished an increase in the effector CD8^+^ T cells in DLN and total and MC38 (Adpgk^MUT^)-specific CD8^+^ T cells in tumor by Bpmel-OVAI-Ea cell adjuvant therapy (Fig. 3 D; and Fig. S4, E and F). These results indicated that CD4^+^ T cells were essential for CTA-induced antitumor effects.

### DCs are the population that exhibits the most significant changes in cross talk signaling after receiving cell adjuvant therapy

CD4^+^ T cells license APCs to promote CD8^+^ T cell activity (Smith et al., 2004). We hypothesized that the cell adjuvant altered the cross talk between different immune cells, consequently altering the activation status of CD8^+^ T cells. To test this hypothesis, scRNAseq was conducted using total DLN cells obtained from MC38 tumor-bearing mice with or without SIY-OVAII. The cells were grouped into 12 clusters based on gene expression (Fig. 3 E). To reveal the interactions across different cell types, we applied CellChat (Jin et al., 2021a) to estimate the probability of their cross talk. By comparing the activities of ligands and receptors (outgoing and incoming signals, respectively) of each cell population before and after the SIY-OVAII treatment, we found that DCs were the cells most affected (Fig. 3 F). To identify which signaling pathways were affected during the treatment, we compared DC activities, as quantified by the communication probabilities of the indicated signals. The strength of signaling in *TIGIT* (immune checkpoint signal) and *CSF* was decreased, and that in *ALCAM*, *CD6*, and *FLT3* (development-supporting signal) in DC was significantly increased after the treatment (Fig. 3 G).

### Cell adjuvant therapy enhances the cross talk between DCs and T cells

**Figure 4.**
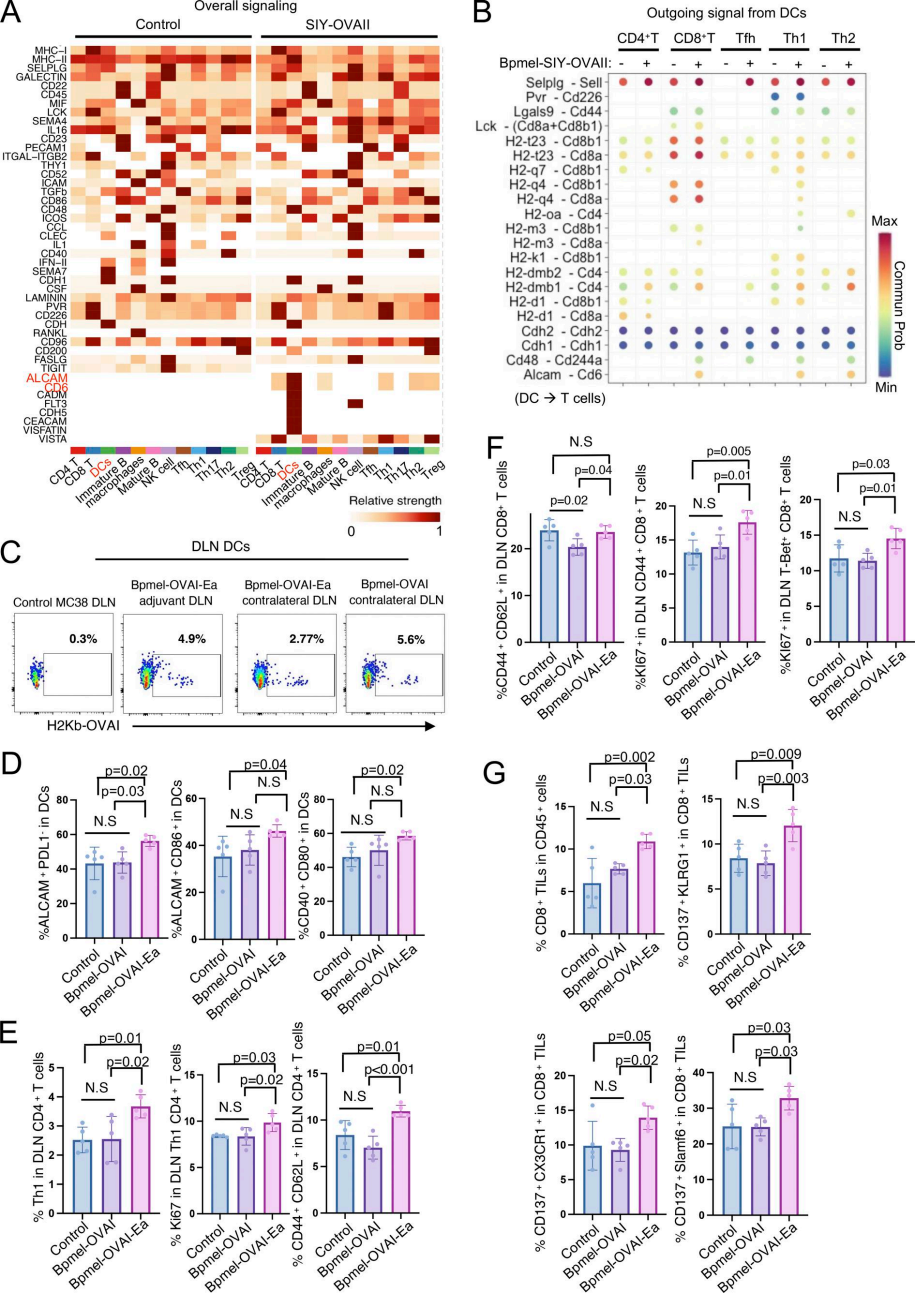
**Complete T cell antigen, but not MHC I–restricted epitope alone, promotes immune cell cross talk and stem-like CD8** ^**+**^ **TIL infiltration. (A and B)** Continuous analysis of the scRNAseq data in Fig. 3; (A) the overall signal strength of each population and signaling pathway; (B) bubble plots indicating the probability of the significant ligand–receptor pairs transferred from DCs to other populations with and without SIY-OVAII cell adjuvant therapy. **(C)** MC38-bearing mice were treated with Bpmel-OVAI-Ea cell therapy. An antibody against H-2kb/OVAI complex was used to detect DCs in DLN that present OVAI antigen. OVAI-presenting DCs were detected in both the ipsilateral and contralateral DLNs 3 days after Bpmel-OVAI-Ea injection. They were also detected in the contralateral DLNs of Bpmel-OVAI. Ipsilateral DLNs from MC38 tumors without cell adjuvant served as a control. **(D–G)** Cell adjuvant therapy of Bpmel-OVAI-Ea or Bpmel-OVAI started on day 6 after the MC38 inoculation (day 0), and the analysis started on day 15; (*n* = 5 per group); (D) flow cytometry analysis of DCs in the MC38-side DLN; (F) flow cytometry analysis of CD8^+^ T cell or CD4^+^ T cells in the MC38-side DLN; (G) flow cytometry analysis of CD8^+^ TILs of MC38. Statistical analysis was performed using one-way ANOVA followed by Dunnett’s multiple comparison. All data are representative of three similar experiments.

To identify which cell type interaction is responsible for the altered signaling in DCs, we further analyzed the scRNAseq data in DLN cells and linked the signaling strength with each cell type. The signaling pathways involving ALCAM, CD6, FLT3, CDH5, and MHC-II were upregulated in DCs from the SIY-OVAII group (Fig. 4 A). After the treatment, there was a general upregulation in the signal through MHC-CD4/8 across DCs and different T cell subsets (CD4^+^, CD8^+^, Tfh, Th1, and Th2) (Fig. 4 B). Notably, the cross talk between DCs and CD8^+^ T cells/T helper cells was significantly enhanced through Alcam–Cd6 axis (Fig. 4 B). This interaction (Alcam–Cd6) between DCs and T cells was necessary for the efficacy of cell adjuvant therapy (Ampudia et al., 2020). Thus, these results indicated that cell adjuvant therapy expressing CTA promoted the cross talk between APCs and T cells, consequently altered the activation status of CD8^+^ T cells.

Since the scRNAseq analysis indicated that DCs were most affected by the cell adjuvant therapy, we further investigated how DCs activate antitumor immunity against MC38. We first examined whether CTA-presenting DCs in the DLN of the cell adjuvant treatment side can traffic to the contralateral DLN of MC38. As shown in Fig. 4 C, in MC38 tumor-bearing mice treated with Bpmel-OVAI-Ea cell adjuvant therapy, DCs presenting OVAI were detectable in both sides of the DLNs, indicating that CTA-presenting DCs traffic beyond their site of injection. However, this migration was also observed in mice treated with Bpmel-OVAI, which did not significantly inhibit tumor growth (Fig. 4 C). We therefore hypothesized that enhanced antitumor immunity is mediated by not the trafficking of DC, but the quality change of DCs as indicated in the scRNAseq analysis above (Fig. 3 F). To validate the scRNAseq data, we performed flow cytometry analysis on MC38-side DLN cells after the treatment with either Bpmel-OVAI or Bpmel-OVAI-Ea. Treatment with Bpmel-OVAI-Ea resulted in a significant increase in ALCAM^+^ PD-L1^−^ and ALCAM^+^ CD86^+^ DCs compared with Bpmel-OVAI, consistent with the scRNAseq findings (Fig. 4 D). Notably, only Bpmel-OVAI-Ea, but not Bpmel-OVAI, induced the generation of CD40^+^ CD80^+^ DCs in the DLN, suggesting that the CTA component facilitates DC activation, whereas MHC I–restricted antigens alone are insufficient to elicit this effect (Fig. 4 D).

Functionally, Bpmel-OVAI-Ea treatment promoted robust CD4^+^ and CD8^+^ T cell responses in the DLN, including increased proportion and proliferation of Th1 cells in DLN, expansion of CD4^+^ and CD8^+^ memory T cells in DLN, and enhanced proliferation of CD44^+^ or T-bet^+^ CD8^+^ T cells in DLN—all effects absent in the Bpmel-OVAI group (Fig. 4, E and F).

Consistent with enhanced T cell priming, Bpmel-OVAI-Ea significantly increased CD8^+^ TIL infiltration. Moreover, higher proportions of CX3CR1^+^ CD137^+^ and CD137^+^ KLRG1^+^ CD8^+^ TILs were observed, indicative of heightened cytotoxic potential. Strikingly, we also detected a significant enrichment of CD137^+^ Slamf6^+^ CD8^+^ TILs—a phenotype associated with stem-like, proliferative T cells. These findings suggest that, unlike MHC I–restricted antigens alone, CTA vaccination fosters a pool of stem-like cytotoxic T cells, which may underlie the enhanced responsiveness to anti–PD-L1 therapy (Fig. 4 G).

### Notch^MUT^ is an MC38-derived natural complete T cell antigen

**Figure 5.**
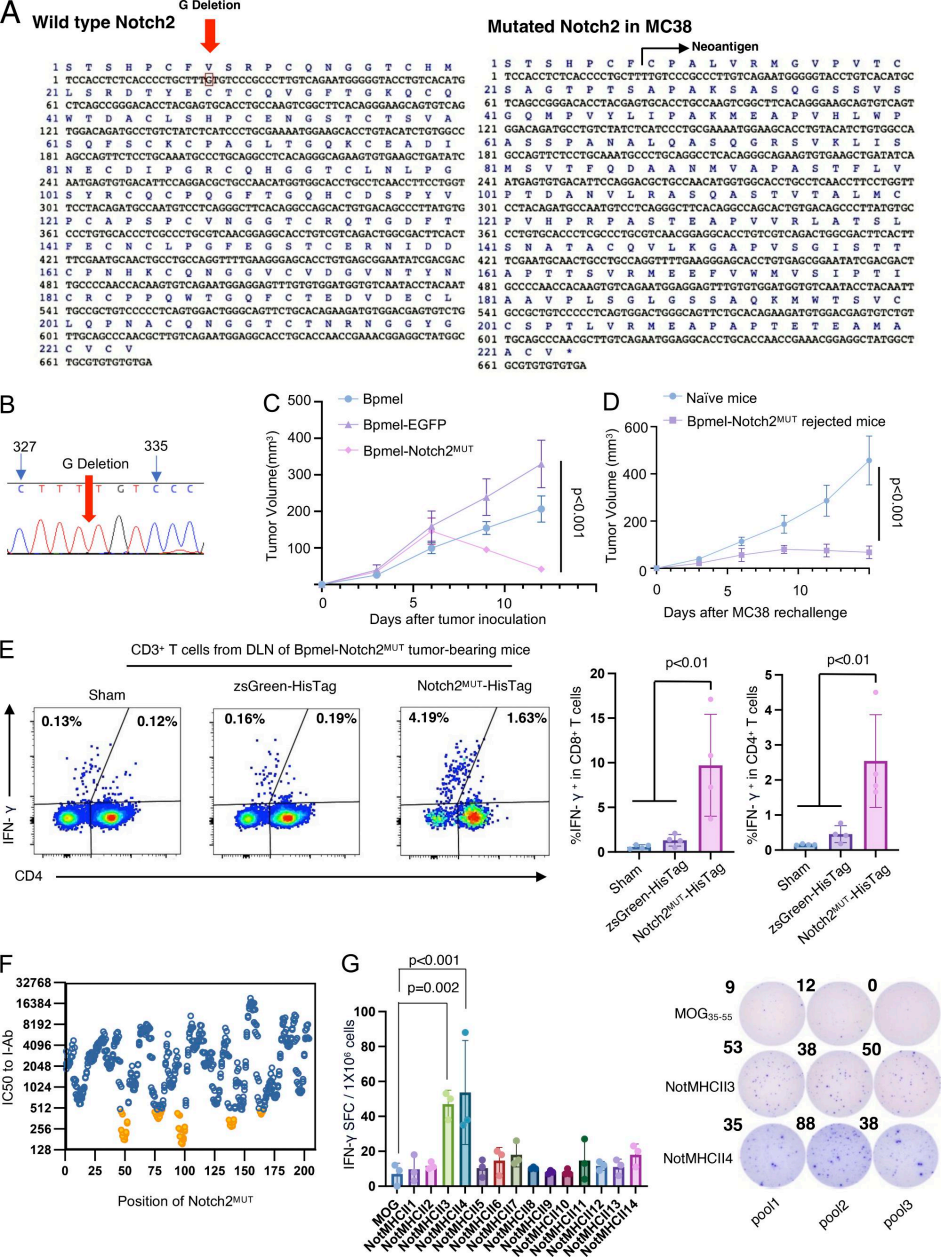
**Notch** ^**MUT**^ **, a long neoantigen, is an endogenous complete T cell antigen of MC38. (A)** The DNA sequence of WT Notch2 (left). The red arrow indicates the region deleted by the mutation in MC38. DNA sequences of mutated Notch2 in MC38 cells were obtained through WES (right). **(B)** The sequence of the MC38 Notch2 cDNA. **(C)** Tumor volumes in mice treated with Bpmel-EGFP or Bpmel-Notch2^MUT^ (*n* = 5 per group). **(D)** MC38 tumor volumes in naïve or Bpmel-Notch2^MUT^–rejected mice (*n* = 5 per group). **(E)** Percentage of IFN-γ–releasing CD4^+^ or CD8^+^ T cells after zsGreen-HisTag or Notch2^MUT^-HisTag stimulation for 16 h (*n* = 4 mice per group). CD3^+^ CD4^−^ T cells were identified as CD8^+^ T cells. **(F)** MHC II peptide-binding map of Notch2^MUT^. The binding affinity of Notch2^MUT^ peptides spanning the Notch2^MUT^ sequence was predicted in silico and expressed as IC50 to I-Ab using hmMHC. The candidate peptide used for verification is marked in yellow. **(G)** Sorted CD4^+^ T cells from the DLN of Bpmel-Notch2^MUT^ tumor-bearing mice were co-cultured with DCs isolated from naïve mice and stimulated with the indicated peptides for 24 h. The left panel shows the number of IFN-γ–producing spot-forming cells (SFC) following peptide stimulation (*n* = 3 per group). The right panel displays three representative wells from the IFN-γ ELISPOT assay. All data are representative of three similar experiments. Statistical analysis was performed using one-way ANOVA followed by Dunnett’s multiple comparison.

**Figure S5.**
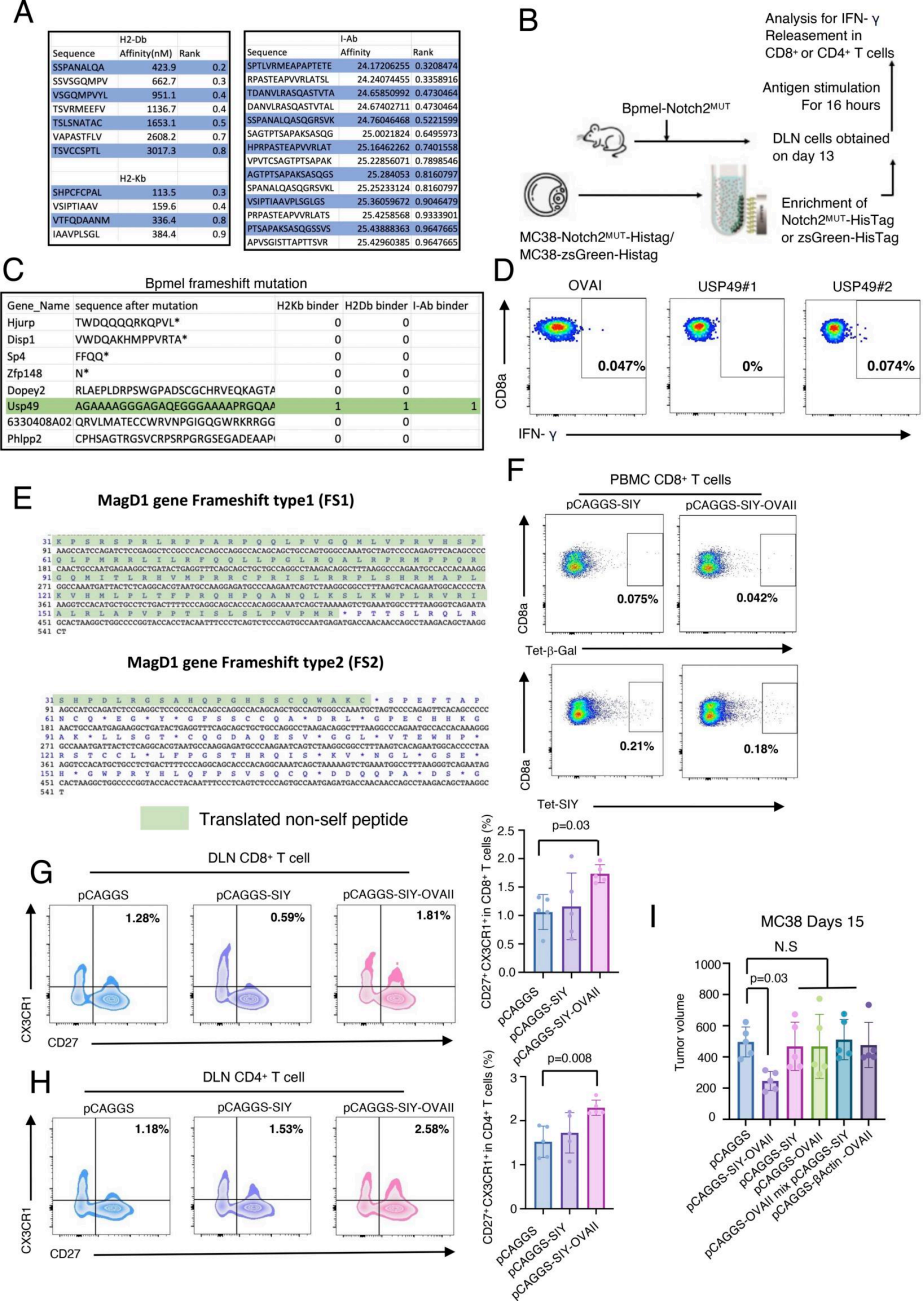
**CTA regulates antitumor immunity through phenotypic changes in T cells. (A)** Predicted affinity of Notch2MUT-derived potent epitopes for H2-Kb and H2-Db (left) and for I-Ab (right). The NetMHCpan algorithm and the HMM-based binding predictor hmMHC were used for analysis. **(B)** The experimental procedure for ex vivo antigen stimulation. 13 days after Bpmel-Notch2MUT injection, DLN cells were stimulated with zsGreen-HisTag or Notch2MUT-HisTag protein that was purified from MC38 cells overexpressing zsGreen-HisTag or Notch2MUT-HisTag. **(C)** All frameshift mutations in Bpmel cells were identified using WES. Sequencing results are listed in Table S5. **(D)** DLNs were isolated from Bpmel tumor–bearing mice on day 13. Cells were stimulated for 6 h with the two predicted MHC I epitopes of USP49, and IFN-γ release was detected (right panels). **(E)** Translated non-self peptide after frameshift 1 knock-in or frameshift 2 knock-in on MagD1 gene locus. **(F)** SIY-specific CD8^+^ T cells were detected using the SIY tetramer 1 wk after electrotransfection; the β-galactosidase tetramer was used as a negative control. **(G and H)** FCM analysis of CX3CR1 and CD27 expression in CD8^+^ or CD4^+^ T cells in DLNs of MC38 tumor-bearing mice 15 days after tumor inoculation (*n* = 5 per group). **(I)** Tumor volumes in MC38-bearing mice electrotransfected with Bpmel-β-actin-OVAII, pCAGGS-SIY, pCAGGS-OVAII, or a mixture of pCAGGS-SIY and pCAGGS-OVAII, or pCAGGS-SIY-OVAII. Statistical analysis was performed using one-way ANOVA followed by Dunnett’s multiple comparison. All data, except for WES data, are representative of triplicate experiments.

All CTAs used in the cell adjuvant therapies thus far were artificially constructed and overexpressed. Therefore, we investigated whether tumor cells endogenously generate CTAs by analyzing MC38 cells as an example. Most studies on neoantigens have focused on missense variants and numbers of mutations. However, we sought to evaluate frameshift mutations because they could generate longer single-peptide chains theoretically containing both MHC I– and II–restricted epitopes. The WES of MC38 cells revealed 25 frameshift mutations, one of which was a guanine deletion at position 331 of *Notch2* (Notch2^MUT^) that lead to a 216 AA-long neoepitope (Fig. 5 A). This mutation was confirmed in cloned *Notch2*^*MUT*^ genes from MC38 cells (Fig. 5 B). The MHC-binding prediction of Notch2^MUT^ peptides generated 11 and 14 strong binders for MHC I (H2-Db and -Kb) and MHC II (I-Ab), respectively (Fig. S5 A), possibly fulfilling the requirements of a CTA.

To determine the immunoreactivity of Notch2^MUT^, we cloned and expressed it in Bpmel cells. Bpmel-Notch2^MUT^ tumors were highly immunogenic and naturally rejected (Fig. 5 C). The growth of MC38 cells rechallenged into mice that had rejected Bpmel-Notch2^MUT^ tumors was markedly suppressed, suggesting memory formation by the Notch2^MUT^ antigen (Fig. 5 D). Furthermore, both CD8^+^ and CD4^+^ T cells from the DLN of Bpmel-Notch2^MUT^ tumor-bearing mice produced IFN-γ in response to stimulation with Notch2^MUT^ protein (Fig. S5 B and Fig. 5 E). These results indicated that the endogenously encoded Notch2^MUT^ in MC38 cells could be recognized by immune cells. To further investigate whether the Notch2^MUT^-derived epitopes were recognized by CD4^+^ T cells, we predicted the binding affinities of candidate epitopes to I-Ab (Fig. 5 F) and synthesized the 14 epitope peptides with the highest predicted affinities for functional testing (Fig. 5 G and Table S4). Among them, NotMHCII3 and NotMHCII4 effectively stimulated CD4^+^ T cells to produce IFN-γ, as demonstrated by ELISPOT assays.

In contrast, WES analysis of Bpmel revealed eight frameshift mutations. Of these, only Usp49 was predicted to be a CTA (two binders for MHC I and one for MHC II) (Fig. S5 C and Table S5). However, neither of the predicted MHC I binders were sufficiently recognized by CD8^+^ T cells (Fig. S5 D). These results suggested that Bpmel generated few CTAs, which may explain its unresponsiveness to anti–PD-L1 therapy. Overall, these results show that MC38 cells encode Notch2^MUT^, endogenously generating a “natural” CTA.

### MC38-derived Notch2^MUT^ reverses the unresponsiveness of tumors to PD-L1 blockade

**Figure 6.**
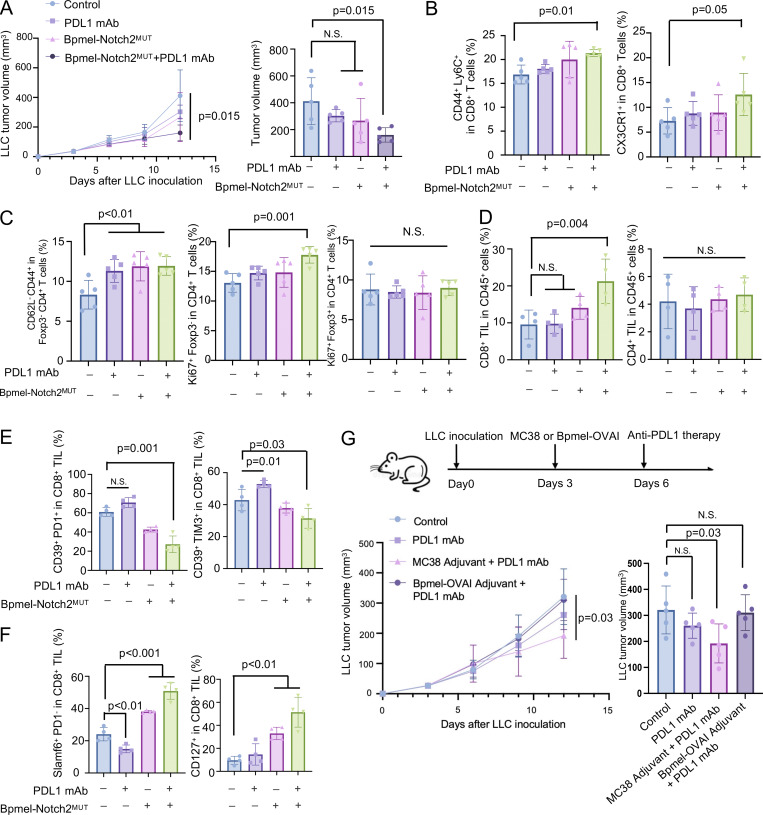
**Artificially or naturally expressed Notch** ^**MUT**^ **restored sensitivity to anti–PD-L1. (A)** Tumor volume in LLC-bearing mice following anti–PD-L1 mAb, Bpmel-Notch2^MUT^ cell adjuvant (Bpmel-Notch2^MUT^), or combination therapy (Bpmel-Notch2^MUT^ + PD-L1 mAb) (*n* = 5 per group). **(B)** The percentage of central memory (CD44^+^Ly6C^+^) and CX3CR1^+^ population in CD8^+^ T cell in DLN. **(C)** The percentage of effector memory (CD62L^−^CD44^+^) among Foxp3^−^ CD4^+^ T cells, Ki67^+^ Foxp3^−^ cells in CD4^+^ T cells, and Ki67^+^ Foxp3^+^ cells in CD4^+^ T cells in DLN. **(D)** The percentage of CD8^+^ or CD4^+^ T cells among the CD45^+^ population in LLC tumors (*n* = 4 per group on day 12). **(E and F)** FCM analysis of CD39, PD1, TIM3, SLAMF6, and CD127 expression in tumor-infiltrating CD8^+^ T cells on day 12 (*n* = 4 per group). **(G)** The experimental procedure of anti–PD-L1 and cell adjuvant combination therapy (upper panel). LLC tumor volume in mice treated with MC38 as a cell adjuvant or Bpmel-OVAI as a negative control combined with anti–PD-L1 antibody is shown (*n* = 5 per group). P value was determined using one-way ANOVA followed by Dunnett’s multiple comparisons test. All data are representative of three similar experiments.

As a cell adjuvant with artificial CTA suppressed the generation of exhausted CD8^+^ T cells and induced memory-like T cells (Fig. 2), we investigated whether the endogenous CTA had a similar effect on CD8^+^ T cell phenotypes and whether such phenotypic changes altered tumor unresponsiveness to the PD-1 blockade therapy. To this end, we performed a combination therapy on mice bearing Lewis lung carcinoma (LLC) tumors, which typically do not respond to anti–PD-L1 treatment (Kumar et al., 2020). Although injection of Bpmel-Notch2^MUT^ cell adjuvant or anti–PD-L1 antibody alone marginally reduced LLC tumor volume, combination treatment with Bpmel-Notch2^MUT^ cell adjuvant and anti–PD-L1 antibody substantially controlled the LLC tumor volume (Fig. 6 A). In the LLC-DLNs, the combination therapy significantly increased the proportions of memory (CD44^+^ Ly6C^+^) and CX3CR1^+^ CD8^+^ T cells, whereas anti–PD-L1 antibody alone had no such effects (Fig. 6 B). All three treatments elevated the proportion of effector CD4^+^ T cells (CD62L^−^ CD44^+^ Foxp3^−^) in the DLNs; however, only the combination therapy enhanced the proliferation of conventional CD4^+^ T cells (Ki67^+^ Foxp3^−^) without affecting the proliferation of Tregs (Ki67^+^ Foxp3^+^) (Fig. 6 C). In the TME, combination therapy increased the infiltration of CD8^+^ TILs but did not affect that of CD4^+^ TILs (Fig. 6 D). Although anti–PD-L1 therapy alone promoted the exhaustion of CD8^+^ TILs and decreased the infiltration of self-replicable slamf6^+^ CD8^+^ TILs, combination therapy partially reversed these effects (Fig. 6, E and F). In addition, the MC38 cell adjuvant enhanced the efficacy of PD-L1 blockade therapy against LLC cells, suggesting that the endogenously expressed Notch2^MUT^ enhanced antitumor immunity (Fig. 6 G). These results indicated that the cell adjuvant with natural CTA could enhance the effect of anti–PD-L1 therapy.

**Figure 7.**
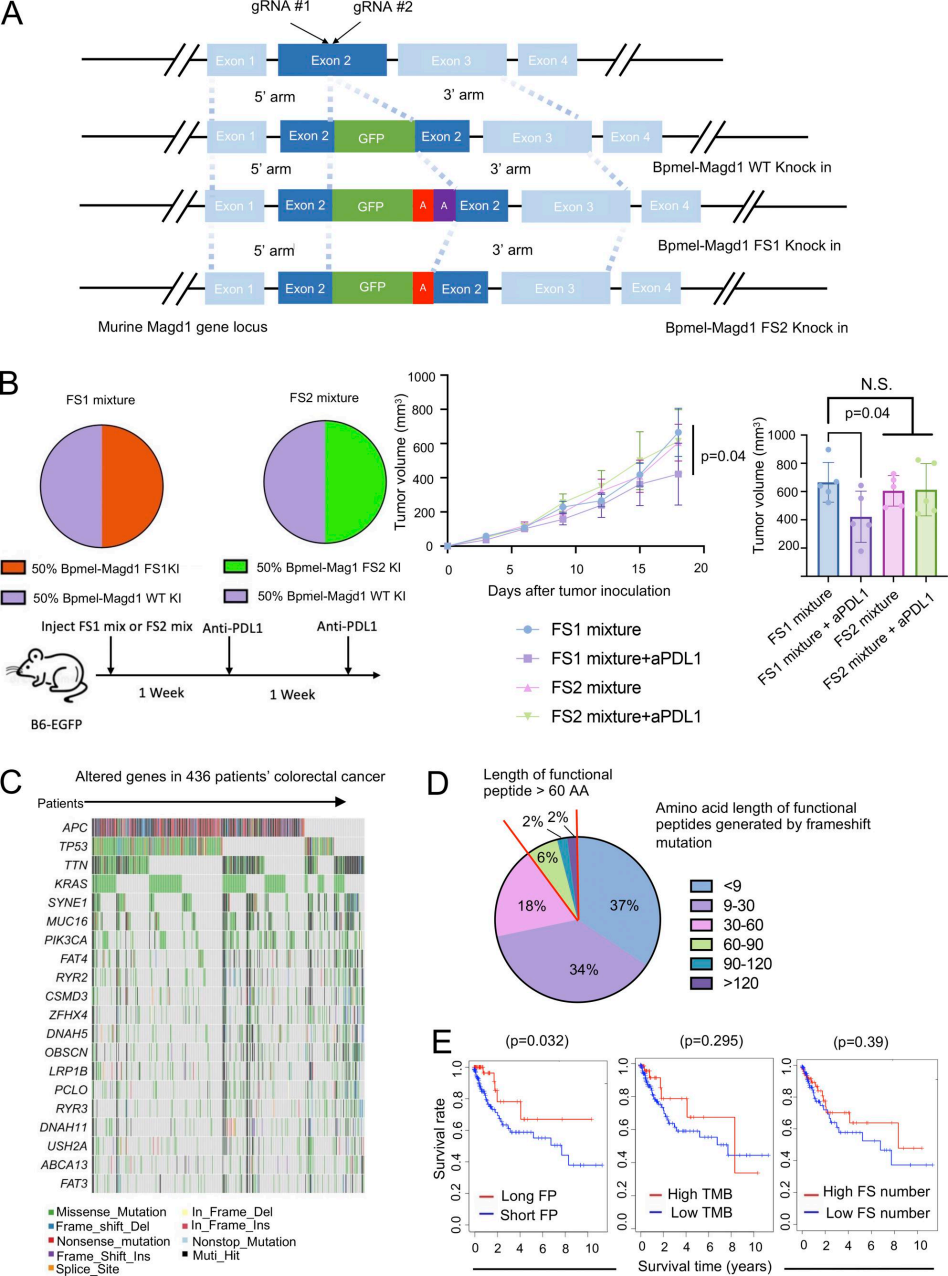
**Long neoantigens generated by frameshift mutation are associated with good** **prognosis** **in patients with cancer. (A)** Genomic organization of the WT and targeting vectors for the murine Magd1 gene. Bpmel-Magd1 WT knock-in was generated by inserting GFP, while Bpmel-Magd1 FS1 knock-in was generated by inserting GFP along with 2 adenines to induce a downstream type 1 frameshift (FS1). Similarly, Bpmel-Magd1 FS2 knock-in was generated by inserting GFP plus 1 adenine to induce a downstream type 2 frameshift (FS2). **(B)** The experimental procedure for anti–PD-L1 therapy in cell mixture tumors (left panel). The FS1 mixture was created by combining 50% Bpmel-Magd1 FS1 knock-in cells with 50% Bpmel-Magd1 WT knock-in cells, while the FS2 mixture was generated by mixing 50% Bpmel-Magd1 FS2 knock-in cells with 50% Bpmel-Magd1 WT knock-in cells. The right panel shows the tumor volume of the cell mixture following anti–PD-L1 therapy (*n* = 5 per group). P value was determined using one-way ANOVA followed by Dunnett’s multiple comparisons test. **(C)** Waterfall plot for the top 20 mutated genes in 436 colorectal cancer patients based on the TCGA dataset. **(D)** Percentages of different lengths of frameshift-generated functional peptides in the patients. **(E)** Kaplan‒Meier analysis of the overall survival of patients whose maximal length of frameshift-generated functional peptides was longer (long FP) or shorter (short FP) than 120 AA (left). FS, frameshift. The top 20% were defined as having high TMB or FS numbers, corresponding to the frequency of patients with long functional peptides. The Kaplan‒Meier method was used to compare two groups of clinical variables. Experiments in B were performed three times.

To further investigate whether the presence of tumor cells expressing only one natural CTA is sufficient to alter overall ICI responsiveness, either a type 1 or type 2 frameshift mutation was introduced by knocking in 2 or 1 nucleotides, respectively, at the murine melanoma-associated antigen D1 (Magd1) gene locus (Fig. 7 A). This gene was selected due to the following two reasons: (1) MagD1 is among top 1% of high expression gene in Bpmel; (2) the type 1, but not the type 2, frameshift mutation generates a significantly longer non-self peptide, predicted to function as a CTA (Fig. S5 E). Tumors with 50% of cells harboring a type 1 frameshift mutation in the Magd1 gene exhibited a stronger response to ICI therapy compared with those with 50% of cells containing a type 2 frameshift mutation (Fig. 7 B). Because cancer tissue is composed of heterogeneous clones, our findings suggest that even if only a subset of these clones express natural CTAs, this may influence the responsiveness of the entire tumor mass to immunotherapy.

### Long neoantigens generated by frameshift mutations are associated with good immune responses in patients with cancer

Accumulated evidence indicates that the prognosis of patients with cancer is strongly associated with immune responses. A good prognosis is related to the high amount of tumor-infiltrated CD8^+^ T cells and natural killer cells (Galon et al., 2006; Pagès et al., 2010; Fridman et al., 2012). We therefore investigated whether CTAs produced in patient tumors also affected patient prognosis. We examined frameshift mutations derived from insertions or deletions (InDel) because these types of genetic abnormalities are highly likely to generate long neopeptides (Xie et al., 2023). We analyzed WES data from a public The Cancer Genome Atlas (TCGA) dataset of 436 patients with colorectal cancer treated with any therapy to analyze InDels. Although the most frequent mutations that theoretically generated neoantigens were missense mutations (78%), InDels accounted for 12% of detected mutations (Fig. 7 C).

Frameshift mutations resulting from InDels usually produce a premature stop codon, preventing further elongation of non-self peptides. Approximately 90% of the non-self peptides produced by InDel-derived frameshift mutations contained fewer than 60 AAs in this cohort (Fig. 7 D). We referred to neopeptides predicted to be generated by frameshift mutations starting from the mutation site to aberrantly generated stop codons as “functional peptides.” Because the length of functional peptides is directly associated with the possibility of CTA generation, we determined whether the maximum length of functional peptides generated by each patient was related to their prognosis. As shown in Fig. 7 E, patients whose tumors encoded functional peptides longer than 120 AA had a significantly better prognosis than those whose tumors encoded those shorter than 120 AA. Notably, the maximum functional peptide length was a better prognostic marker than the TMB or number of frameshift mutations (Fig. 7 E). These results suggest that long frameshift-derived peptides may regulate cancer immunoreactivity, which may be attributed to CTA generation.

### Intramuscular electrotransfection of a CTA vector enhances antitumor immunity

**Figure 8.**
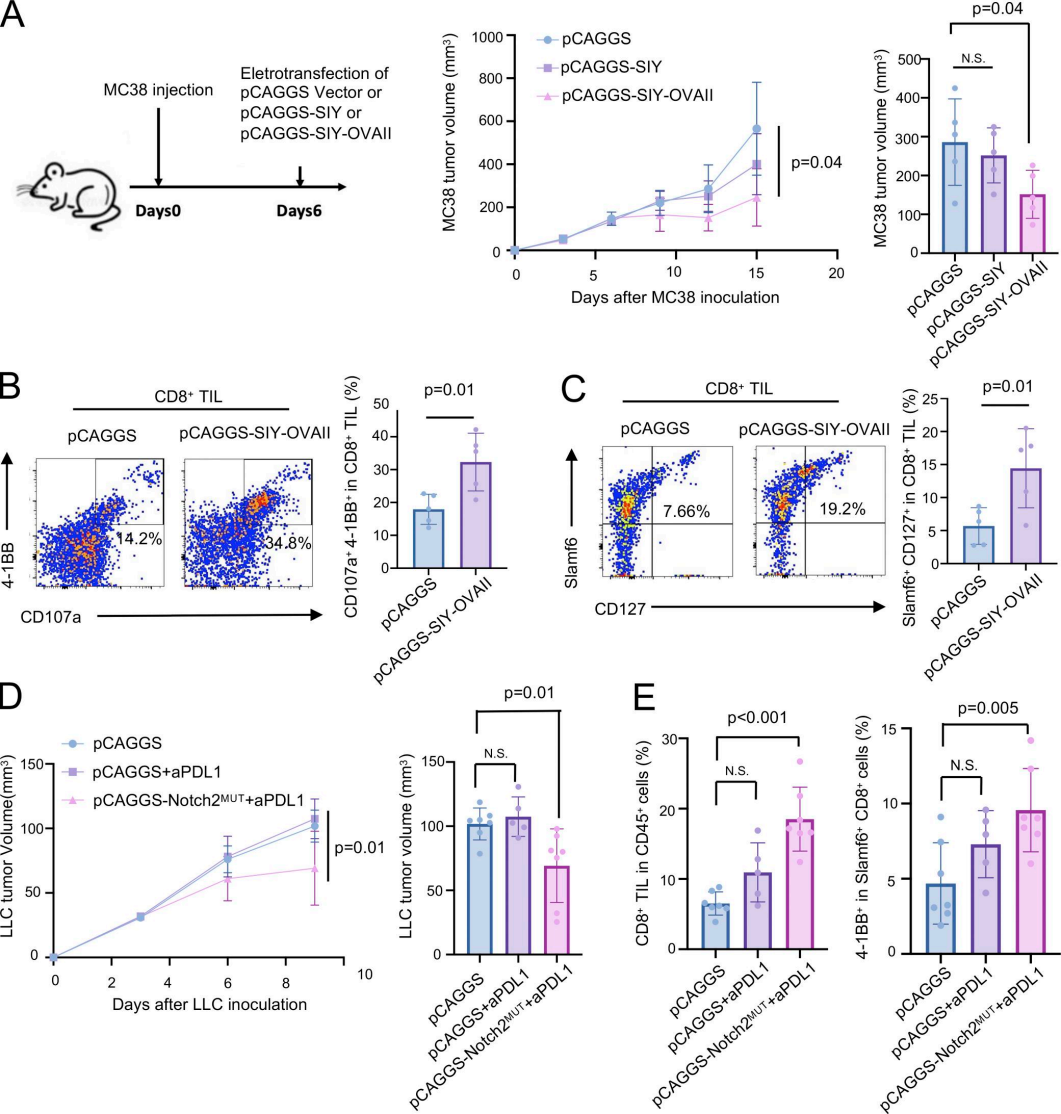
**The intramuscular introduction of CTAs through electrotransfection systemically enhances antitumor immunity. (A)** The experimental procedure for cDNA-based immunization with pCAGGS-SIY-OVAII (left); pCAGGS-SIY was used as a negative control. The volume of MC38 tumors after electrotransfection of pCAGGS, pCAGGS-SIY, or pCAGGS-SIY-OVAII in the quadriceps (right) (*n* = 5 per group). **(B and C)** FCM analysis of 4-1BB, CD107a, CD127, and SLAMF6 expression in tumor-infiltrated CD8^+^ T cells on day 15 (*n* = 5 per group). **(D)** The volume of LLC tumors after electrotransfection of pCAGGS or pCAGGS-Notch2^MUT^ with or without anti–PD-L1 therapy in the quadriceps (*n* = 5 per group). **(E)** FCM analysis of CD8^+^ T cell infiltration and 4-1BB expression in tumor-infiltrating SLAMF6^+^ CD8^+^ T cells on day 10 (*n* = 5 per group); statistical analysis was performed using Student’s *t* test (B and C) or one-way ANOVA followed by Dunnett’s multiple comparisons test (A, D, and E). All data are representative of two to three similar experiments.

In clinical settings, it is challenging to inoculate patients with CTA-expressing cell lines as adjuvants. Therefore, we investigated whether CTA expression in muscle cells through the electrotransfection of DNA vectors could enhance antitumor immunity. One week after MC38 inoculation, pCAGGS-SIY-OVAII was electrotransfected into the contralateral quadriceps (Fig. 8 A). SIY-specific CD8^+^ T cells were detected in PBMCs on day 14, indicating successful transfection and immunization (Fig. S5 F). Only the electrotransfection of pCAGGS-SIY-OVAII, but not pCAGGS-SIY, promoted antitumor immunity and the generation of the progenitor-like CD27^+^ CX3CR1^+^ cytotoxic population in CD8^+^ and CD4^+^ T cells in the DLN (Fig. 8 A; and Fig. S5, G and H) (Zwijnenburg et al., 2023). Electrotransfection with pCAGGS-SIY-OVAII promoted the infiltration of 4-1BB^+^ CD107a^+^ (activated) and Slamf6^+^ CD127^+^ (self-replicable) CD8^+^ T cells in the tumor (Fig. 8, B and C) (Watanabe et al., 2008; Milner et al., 2020). Electrotransfection of Bpmel-β-actin-OVAII, pCAGGS-SIY, pCAGGS-OVAII, or a mixture of pCAGGS-SIY and pCAGGS-OVAII did not exhibit any antitumor effect, further supporting our previous conclusion (Fig. S5 I). Similarly, electrotransfection of pCAGGS-Notch2^MUT^ into LLC tumor-bearing mice reversed anti–PD-L1 unresponsiveness (Fig. 8 D) and significantly promoted CD8^+^ TIL infiltration and 4-1BB expression on SLAMF6^+^ CD8^+^ TILs in LLC tumors (Fig. 8 E). These results indicate that electrotransfection of the CTA expression vector promoted antitumor properties and could help reverse unresponsiveness to anti–PD-L1 therapies.

## Discussion

**Figure 9.**
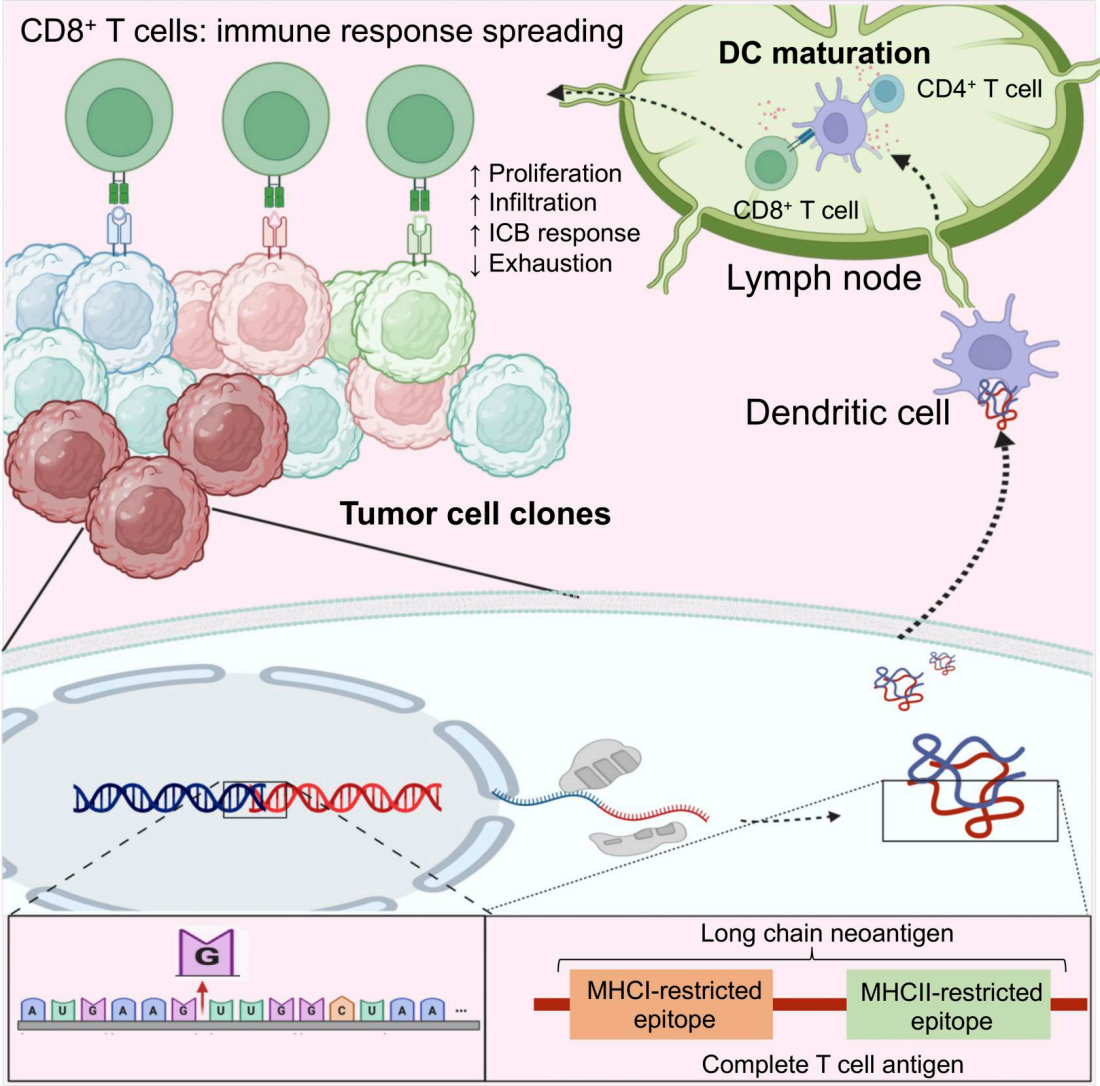
**Proposed mechanism for the CTA-induced antitumor effect.** Tumor cells may generate CTAs through frameshift mutations. CTA is subsequently presented by DCs and enhances cross talk with CD8^+^ and CD4^+^ T cells. This DC–T cell cross talk spreads the T cell immune responses to previously unrecognized neoantigens with low immunoreactivity, leading to better ICB response and prognosis.

This study identified the immunologically meaningful neoantigen for the activation of overall antitumor immunity, particularly those being chimeric for at least one MHC I– and one MHC II–restricted non-self epitopes in a single chain, called CTA (Fig. 9). Even when CTA is neoantigen unrelated, CTA therapy trigger the chain reaction of CD8^+^ T cells to various less immunoreactive neoantigens, inducing broad spectrum of antitumor effects. The intracellular expression of artificial CTAs systemically enhances the efficacy of PD-L1 blockade therapy in vivo. Because long neopeptides increase the chance of CTA generation, the length of neopeptides in cancer cells is associated with their immunoreactivity and responsiveness to PD-1 blockade therapy in mouse and/or human studies. Our results indicate that if some cancer cells within a tumor mass express CTAs, the overall antitumor immunity against different neoantigens would be enhanced.

The effect of CTA therapy is CD4^+^ T cell–dependent. CTAs may boost the “cognate help” of CD4^+^ T cells, where CD4^+^ and CD8^+^ T cells recognize antigens on the same DCs. In agreement with our results, the immune triads formation among CD4^+^ T cells, CD8^+^ T cells, and DCs in tumor is reported to be critical for tumor elimination (Espinosa-Carrasco et al., 2024). Cognate help facilitates the breakdown of cytotoxic lymphocyte tolerance and the full induction of immunity by licensing DCs (Smith et al., 2004; Thaiss et al., 2011; Steinaa et al., 2005). CTA treatment alters the features of DCs in the DLN, with enhanced expression of co-stimulatory signals related to the CD86, MHC II, and CD40 pathways. Moreover, the co-stimulation signal CD6-ALCAM in DCs, which plays a vital role in the restimulation of effector T cells, is only detected after CTA therapy (Zimmerman et al., 2006; Ibáñez et al., 2006). These findings suggest that phenotypically altered DCs may induce Slamf6^+^ progenitor T cells responsive to ICI, which function as reservoir cells for effector CD8^+^ TILs (Connolly et al., 2021). However, more detailed analysis of DC phenotype alterations is necessary.

Several groups reported that the expression of MHC II–restricted antigens by tumor cells is required at the site of effective immunotherapy (Alspach et al., 2019; Espinosa-Carrasco et al., 2024). We also confirmed the critical role of MHC II–restricted antigens and CD4^+^ T cells for the DC licensing in our model. However, our findings differ in several key aspects: First, CTA expression is not required within the TME, giving it a unique potential for therapeutic application. Second, CTA-induced DC cross talk primarily occurs in the DLN, rather than in the TME. Third, the antitumor effect of CTA can extend T cell responses across antigen specificities.

Genomic, transcriptional, and translational errors can generate long non-self antigens (functional peptides) in cancer cells. Although point mutations are the most frequent type of mutation at the genomic level, the products of point mutations are insufficient for CTA generation (Xie et al., 2023). Gene fusions can produce long functional peptides, but they are very rare (Wang et al., 2021). In contrast, InDel-derived frameshift mutations can generate long functional peptides with more frequency than gene fusions (Xie et al., 2023). At the transcriptional and translational levels, several mechanisms can lead to frameshifts, including ribosomal frameshifting, transcriptional slippage, transcription infidelity, transcriptional RNA editing, and aberrant RNA splicing (AS) (Xie et al., 2023). As AS is usually caused by mutations in splice sites or splicing regulatory elements, it is less accidental than other mechanisms. In the analysis of the database of colon cancer patients, we identified the length of neoantigens but could not estimate the reactive epitopes due to the lack of HLA type information. Therefore, it is a limitation to know whether their long functional peptides really contain chimeric peptides in this human study.

Beyond tumor cells, infection can contribute to the generation of CTA and be associated with cancer therapeutic effects. Since the 1930s, intravesical Bacillus Calmette-Guérin has been used as a cancer therapy for superficial bladder cancer (Meyer et al., 2002). More recently, SARS-CoV-2 infection has been reported to promote lymphoma remission (Kurlapski et al., 2022; Challenor and Tucker, 2021). Our previous findings also demonstrated that xenogeneic or allogenic cell treatment induces an antitumor effect (Nakajima et al., 2021)—those observations were challenging to explain within the framework of classical immunology, given the rarity of cross-reactivity at the epitope level. The CTA theory provides a compelling and rational hypothesis for these phenomena, offering a new perspective on the underlying immunological mechanisms.

Personalized neoantigen immunization has received increased attention as a novel cancer immunotherapy (Finn and Rammensee, 2018; Rojas et al., 2023). The basic principle of these therapies is to identify neoantigens specific to a patient’s tumor and immunize the patient based on the corresponding epitopes to promote neoantigen-specific CD8^+^ T cell generation. However, the clinical effects of these therapeutic strategies are usually unsatisfactory, even if they induce neoantigen-specific T cell responses (Cafri et al., 2020; Awad et al., 2022; Ding et al., 2021). The inclusion of MHC II–restricted epitopes is required to maximize the effect of antitumor vaccines (Aarntzen et al., 2013). Our results suggest several points that may be worth noting during the development of neoantigen vaccines: (1) MHC II–restricted epitopes should be incorporated into the same peptide chain as MHC I–restricted epitopes; (2) intracellular expression, such as mRNA-based delivery, may induce a more substantial antitumor effect than peptide immunization; and (3) selected epitopes do not need to be the same as tumor neoepitopes. Personalized neoantigen vaccines are specific to individual patients, making them difficult to popularize. In contrast, our CTA therapy can induce broad-spectrum antitumor immunity without knowing the exact neoantigen sequence in each patient. However, the CTA sequence needs to be customized based on the HLA type of each patient because T cell epitopes vary with HLA types (Sidney et al., 2020).

Our study demonstrates that tumor cells that generate CTAs, even only in some of these cells, enhance CD8^+^ T cell responses to various cancer antigens. This type of neoantigen alters DC phenotypes to induce proliferative CD8^+^ T cells reactive to other neoantigens in a CD4-dependent manner. The intracellular expression of these non-self chimeric antigens, even unrelated to tumor antigens, overcomes the immune checkpoint therapy resistance in vivo. Thus, further investigation of CTA will facilitate the development of novel cancer immunotherapies.

## Materials and methods

### Mice and cells

The mice were maintained under specific pathogen-free conditions at the Institute of Laboratory Animals, Graduate School of Medicine, Kyoto University, Kyoto, Japan. All animal experiments were approved by the Animal Research Committee of the Graduate School of Medicine, Kyoto University, Kyoto, Japan, and conducted in accordance with its guidelines. C57BL/6 N WT and C57-Tg (CAG-EGFP) mice were purchased from Charles River Laboratories (Japan) and Japan SLC, respectively. The murine colon adenocarcinoma (MC38) cell line was provided by J.P. Allison (Memorial Sloan-Kettering Cancer, New York City, NY, USA), and Bpmel cells were derived from spontaneously developed melanoma in gene-engineered mice (B6.Cg-Braf^tm1Mmcm^ Pten^tm1Hwu^ Tg[Tyr-cre/ERT2]13Bos/BosJ) with BRAF-mutated/PTEN-loss genotype (Nakamura et al., 2024). Bpmel rarely express MHC II. LLC cells were obtained from American Type Culture Collection. APCs, including Bpmel-OVA, Bpmel-OVAI-Ea, MC38-OVAI-Ea, Bpmel-SIY-OVAII, B-OVAI, Bpmel-Adpgk^MUT^-OVAII, and Bpmel-OVAII, were generated by transducing tumor cell lines with a pLJM1-based lentivirus followed by limiting dilution, and 5 μg/ml puromycin was used to select and maintain transduced cells. The cells were maintained in RPMI 1640 (11875-093; Gibco) or Dulbecco’s modified Eagle medium (11995-065; Gibco) supplemented with 10% heat-inactivated fetal bovine serum and penicillin–streptomycin (26253–84; NACALAI TESQUE) and were free of mycobacterial infection. The cell cultures were maintained at 37°C with 5% CO_2_ in a humidified incubator.

### Mouse therapy models

MC38 (2 × 10^6^), Bpmel cells (1 × 10^6^), and LLC (1 × 10^6^) cells were intradermally (i.d.) injected into the left flank of mice (day 0). Tumor growth was monitored by measuring the tumor size using calipers, and the volume was calculated using the formula for a typical ellipsoid: π (length × breadth × height)/6. For the cell adjuvant model, antigen-expressing tumor cells (1 × 10^6^), which would be naturally rejected, were i.d. injected into another flank 6 days after tumor cell inoculation unless indicated. Protein-based immunization was performed using CFA-emulsified (1:1) OVA.

For electrotransfection of the CTA-encoded cDNA vaccine, mice were intramuscularly administered the plasmid, followed by in vivo electroporation as described previously (Yato et al., 2023). Briefly, hyaluronidase (50 U) was injected into the quadriceps. After 15 min, the mice were anesthetized with isoflurane, and 50 μg of pCAGGS, pCAGGS-SIY, pCAGGS-SIY-OVAII, or pCAGGS-Notch2^MUT^ was injected into the quadriceps. Electrode needles were then inserted into the muscle, and electric pulses were delivered using an electric pulse generator (NEPA21; Nepa Gene). Three poring pulses (50 V, 30 ms), followed by three transfer pulses (20 V, 50 ms), were applied to each injection site.

### Cell preparation for analysis

Axillary, brachial, and inguinal LNs were harvested from the tumor-bearing side (left side) of the mice for DLN analysis. For TIL analysis, tumor tissues were harvested and cut into 1–2-mm pieces with scissors, followed by digestion with collagenase type IV (catalog #LS004188; Worthington Biochemical Corporation) using a gentle MACS Dissociator (Miltenyi Biotec).

### Establishment of Magd1 knock-in cells

Top 1% high expression gene of Bpmel cell was selected based on RNA-seq. We finalized the Magd1 gene and the insertion site by computationally simulating the insertion of one to two nucleotides. Through these simulations, we compared the functional peptides produced by frameshift types 1 and 2, aiming to maximize the difference of length between them.

We designed three gene-targeting vectors with GFP and zero to two nucleotides to disrupt the Magd1 gene in the Bpmel cell line by the CRISPR/Cas9 technique with the double nicking method. PCR fragments used to target the vector were assembled with the GeneArt Seamless Cloning and Assembly Kit (Thermo Fisher Scientific). Oligos encoding guide RNAs were cloned into pX335-U6-Chimeric_BB-CBh-hSpCas9n (D10A) (Addgene). Gene-targeting procedures have been previously described (Ran et al., 2013), and GFP^+^ knock-in cells were sorted by FACSAria (BD Biosciences).

### Flow cytometric analysis and tetramer preparation

The following mAbs were used to detect the indicated antigens: CD3 (145-2C11) and CD8 (53-6.7) (Invitrogen, Carlsbad, CA, USA); CD137 (17B5), CD107a (W18263B), CD44 (IM7), CD62L (MEL-14), CD127 (A7R34), KLRG1 (2F1), CX3CR1 (QA16A03), CD27 (LG.3A10), CD39 (Duha59), PD1 (29F.1A12), TIM3 (B8.2C12), and Slamf6 (330-AJ) (BioLegend); CD8 (KT15) (MBL Life Science); and CD45RB (16A) and CD4 (RM4-4) (BD Pharmingen). The H-2Kb-negative (SIY) tetramer-SIYRYYGL-PE (TS-M008-1) and the H-2Kb OVA tetramer-SIINFEKL-PE (TS-5001-1C) were obtained from MBL Life Sciences, and the H-2Db-Adpgk^MUT^ pentamer was obtained from ProImmune. Neoantigen tetramer construction was performed using the QuickSwitchTM Quant H-2Kb tetramer kit (TB-7400-K1, MBL) with Scap^MUT^ or ARAF^MUT^ peptide (10 μM) according to the manufacturer’s instructions. Flow cytometry was performed using an LSRFortessa X-20 flow cytometer (BD Biosciences), and the data were analyzed using the FlowJo software (FlowJo).

### Epitope prediction

To identify point mutations in Bpmel melanoma cells and predict MHC-I epitopes, all missense mutations in Bpmel melanoma cells were analyzed for their potential to form MHC I epitopes that bind to H-2Db or H-2Kb based on the NetMHCpan algorithm provided by the Immune Epitope Database and Analysis Resource. The results were presented as affinity values (1/IC50 × 100, where IC50 is the half-maximal inhibitory concentration). In Notch^MUT^-related experiments, the prediction of H2-I-Ab–restricted epitopes was based on the HMM-based binding predictor hmMHC, and the top 1% affinity was deemed a strong binder.

### Immune subset depletion and checkpoint blockade

CD4^+^ T cells were depleted through the intraperitoneal administration of 200 μg of depleting antibody (InVivoMAb anti-mouse CD4, clone GK1.5; Bio X Cell) 4 days before therapy. For checkpoint blockade, mice were intraperitoneally injected with 40 μg per dose of α-mouse PD-L1 mAb (clone 1-111A.4) every 6 days.

### Allele-specific PCR

Total RNA was isolated from MC38 cells using Nucleospin RNA (macherey-nagel) and used for cDNA synthesis using ReverTra Ace reverse transcriptase (TOYOBO). The Notch2 segment was amplified using PCR, and the restriction sites Nhel and EcoRI were added (sense, 5′-CGG​CTA​GCA​TGT​CCA​CCT​CTC​ACC​CCT​G-3′; antisense, 5′-CCG​GAA​TTC​CGC​TCC​AGC​CGT​TCA-3′); the gene segment was cloned and inserted into the pLJM1 vector, transformed into competent *Escherichia coli*, and allowed to grow overnight in a selection plate. The presence of Notch2 in each *E. coli* colony was checked with two primers designed to detect the WT allele (sense, 5′-CTC​ACC​CCT​GCT​TTG​TGT-3′; antisense, 5′-GGC​ATC​TGT​AGG​AAC​CAG​GA-3′) and another primer designed to detect the frameshift mutation allele (sense, 5′-CTC​ACC​CCT​GCT​TTT​GTC-3′; antisense, 5′-GGC​ATC​TGT​AGG​AAC​CAG​GA-3′). The Notch2 frameshift mutation was confirmed using DNA sequencing.

### Selective binding and separation of His-tagged protein

MC38-ZsGreen-HisTag or MC38-Notch2MUT-HisTag was lysed by adding 0.5 ml of xTractor Buffer (Z5623N; Takara) per 25 mg of cell pellet, and 1 μl of 1 U/μl DNase I solution was added. The insoluble material was removed after centrifugation at 1,000 × *g* for 15 min at 4°C, and the His-tagged protein was purified using His60 Ni Magnetic Beads (635692; Takara) according to the manufacturer’s instructions. Protein-bound beads were washed twice with His60 Ni equilibration buffer, and His-tagged proteins were obtained by adding elution buffer. The obtained proteins were used for subsequent experiments.

### scRNAseq analysis

The MC38 tumor-bearing mouse model received Bpmel-SIY-OVAII immunization on day 6 after tumor inoculation, and CD45^+^ CD8^+^ TILs were isolated from pooled MC38 tumors (five mice per condition) and sorted 7 days after Bpmel-SIY-OVAII live adjuvant therapy. Naïve T cells (CD3^+^CD44^−^) were removed from pooled DLN single cells using a BD FACSMelody (BD Biosciences), and the remaining DLN cells were used for single-cell sequencing. RNA of these cells were then sequenced using 10x Genomics, and sequencing was carried out using the NextSeq 2000 system. The resulting FASTQ files were processed with Cell Ranger Count using the default settings for 5′ RNA gene expression analysis (Cell Ranger v4.0.0; 10x Genomics).

Unsupervised clustering was performed using the FindNeighbors method implemented in Seurat with default parameters and FindClusters with a resolution of 0.3 (Hao et al., 2021). For supervised analysis and comparison with previous annotations, the scRNAseq data were projected into a reference atlas of TILs using the ProjecTILs package (Andreatta et al., 2021); the lineage trajectory of CD8^+^ TILs was analyzed using the Monocle3 package (Cao et al., 2019), and the CellChat package (version 1.1.3) was used to infer, analyze, and visualize cell–cell communication (Jin et al., 2021). scRNAseq analysis was performed using RStudio (R version 4.3.0; R Foundation for Statistical Computing, Vienna, Austria). The data were deposited in the Gene Expression Omnibus (GEO) repository (https://www.ncbi.nlm.nih.gov/geo/) under accession no. GSE245743.

### TCGA dataset analysis

Colon adenocarcinoma (TCGA-COAD) datasets were downloaded from TCGA data portal (http://tcga-data.nci.nih.gov). We extracted somatic mutation and fragments per kb per million-normalized RNAseq data using the “data matrix” tool provided by the TCGA data portal. The somatic mutation data were processed and analyzed using the “maftools” package in R (version 4.3.0) to explore the mutation landscapes of colon cancer (Mayakonda et al., 2018). The TMB was defined as the total number of somatic mutations, including somatic, insertion-deletion, and coding mutations, as well as base replacements per million bases. For frameshift mutations, the CDS after the mutation site was translated to an AA, and the sequence between the mutation site and the naturally generated stop codon was defined as a “functional peptide.”

Patient-derived colon cancer cells (TCGA) were separated into low- and high-TMB groups according to the median TMB. They were also separated into low- and high-FS groups based on the number of frameshift mutations and into long- and low-FP groups based on whether the patient could generate functional peptides longer than 120 AA. These data were merged with the corresponding clinical information to analyze the correlations between TMB, number of frame shifts, length of functional peptides, and clinicopathological factors in patients with colon cancer. The Wilcoxon rank-sum test was used to compare the two groups of clinical variables.

### Antigen-specific assays

Bpmel or Bpmel-Notch2^MUT^ was i.d. injected into the right flank of the mice, and the mice were allowed to grow for 2 wk. DLN single cells were obtained after 2 wk, cultured (5 × 10^5^ cells/well) in RPMI 1640 supplemented with mouse serum, and stimulated with the indicated peptide (5 μg/ml) for 6 h or stimulated with full-length antigen (10 μg/ml) for 16 h at 37°C with 5% CO_2_ in a humidified incubator. After stimulation, IFN-γ release from CD4^+^ or CD8^+^ T cells was measured using a mouse IFN-γ secretion assay according to the manufacturer’s instructions (130-090-516; Miltenyi Biotec), and peptide synthesis was performed using GenScript.

### ELISpot assay

6-wk-old female C57BL/6 N mice were immunized with Bpmel or Bpmel-Notch2^MUT^. The ELISpot assay was performed using a Mouse IFN-γ ELISpot (ImmunoSpot) according to the manufacturer’s instructions. Freshly isolated DLNs (5 × 10^5^ cells) were co-incubated with peptide overnight at 37°C under 5% CO_2_ in an ELISpot polyvinylidene difluoride white plate coated with an anti–IFN-γ antibody (clone AN18). The H-2Kb OVA peptide SIINFEKL (MBL) was used as a negative control to analyze the MHC I–restricted peptide.

In another experiment, 1 × 10^6^ CD4^+^ T cells isolated from the DLN were mixed with the 1 × 10^5^ CD11c^+^ cells isolated from the spleen; these cells were stimulated with peptides for 24 h, and MOG was used as the negative control.

IFN-γ secretion was detected using a capture antibody (clone R4-6A2) and 5-bromo-4-chloro-3-indolyl phosphate/nitro blue tetrazolium–plus substrate. Spots were then counted using a dissection microscope after the plate was allowed to dry. Spot images were acquired using the ImmunoSpot S6 Ultimate Analyzer (Cellular Technology Limited).

### Statistical analysis

The data were analyzed using Prism 7 (GraphPad Software) and are presented as the mean ± standard deviation (SD). Comparisons between two groups were performed using an unpaired two-tailed Student’s *t* test; comparisons of more than two groups were performed using one-way ANOVA, followed by Dunnett’s multiple comparisons test. Survival analysis was performed using the Kaplan–Meier method, and survival curves were compared using the log-rank test.

### Online supplemental material

Fig. S1 shows that chimeric MHC I– and MHC II–restricted epitopes are essential for complete T cell antigen-induced antitumor effects. Fig. S2 shows the identification of neoantigens of Bpmel. Fig. S3 shows that complete T cell antigen promotes the infiltration of self-replicable CD8^+^ T cells and suppresses their exhaustion in the tumor. Fig. S4 shows the properties of each CD8^+^ TIL cluster. Fig. S5 shows that CTA regulates antitumor immunity through phenotypic changes in T cells. Table S1 shows the epitopes used for the artificial complete T cell antigen. Table S2 shows the sequences of artificial antigen. Table S3 shows the missense mutation epitopes in Bpmel tumor. Table S4 shows the candidates of MHC II–restricted epitope peptides of Notch2^MUT^. Table S5 shows the frameshift-derived functional peptides of Bpmel.

## Supplementary Material

Table S1 shows the epitopes used for the artificial complete T cell antigen.

Table S2 shows the sequence of artificial antigen.

Table S3 shows the missense mutation epitopes in Bpmel tumor.

Table S4 shows the MHC II–restricted candidate epitope peptide of Notch2^MUT^.

Table S5 shows the frameshift-derived functional peptide of Bpmel.

## Data Availability

Single-cell sequence data were deposited in the GEO repository (https://www.ncbi.nlm.nih.gov/geo/) under accession no. GSE245743. The datasets generated and/or analyzed during the current study are available from the corresponding author on reasonable request.
