# Supplementary material for: Chimeric MHC class I– and II–restricted non-self epitopes broaden antitumor T cell reactions
Source: J Exp Med. 2025 Dec 5;223(2):e20250025. doi: 10.1084/jem.20250025 (PMC12679993; doi:10.1084/jem.20250025)
Supplement: Table S1 — shows the epitopes used for the artificial complete T cell antigen. [file jem_20250025_tables1.docx]

**Table S1: The epitopes used for the artificial complete T cell antigen**

| Epitope name | Restricted to | Type | Sequence |
| --- | --- | --- | --- |
| OVAI | H-2Kb | MHC I-restricted | SIINFEKL |
| Adpgk^MUT^ | H-2Db | MHC I-restricted | ASMTNMELM |
| SIY | H-2Kb | MHC I-restricted | SIYRYYGL |
| OVAII | I-Ab | MHC II-restricted | ISQAVHAAHAEINEAGR |
| Eα | I-Ab | MHC II-restricted | ASFEAQGALANIAVD |

Red indicates MHC II–restricted epitopes, and purple indicates MHC I–restricted epitopes.
