# Supplementary material for: Chimeric MHC class I– and II–restricted non-self epitopes broaden antitumor T cell reactions
Source: J Exp Med. 2025 Dec 5;223(2):e20250025. doi: 10.1084/jem.20250025 (PMC12679993; doi:10.1084/jem.20250025)
Supplement: Table S2 — shows the sequence of artificial antigen. [file jem_20250025_tables2.docx]

**Table S2: Sequence of artificial antigen**

| OVAI | SIINFEKLAAYSIINFEKLAAYSIINFEKL |
| --- | --- |
| OVAII | ISQAVHAAHAEINEAGRAAYISQAVHAAHAEINEAGRAAYISQAVHAAHAEINEAGR |
| SIY-OVAII | ISQAVHAAHAEINEAGRAAYISQAVHAAHAEINEAGRAAYISQAVHAAHAEINEAGRAAYSIYRYYGLAAYSIYRYYGLAAYSIYRYYGL |
| OVAI-Ea | ASFEAQGALANIAVDAAYASFEAQGALANIAVDAAYASFEAQGALANIAVDAAYSIINFEKLAAYSIINFEKLAAYSIINFEKL |
| Adpgk^MUT^-OVAII | ISQAVHAAHAEINEAGRAAYISQAVHAAHAEINEAGRAAYISQAVHAAHAEINEAGRAAYASMTNMELMAAYASMTNMELMAAYASMTNMELM |
| β-Actin OVAII | SQAVHAAHAEINEAGRAAYISQAVHAAHAEINEAGRAAYISQAVHAAHAEINEAGRAAYGDEAQSKRAAYGDEAQSKRAAYGDEAQSKR |
| SIY | SIYRYYGLAAYSIYRYYGLAAYSIYRYYGL |
| OVA | MKWVTFISLLFLFSSAYSRGVFRRDAHKSEVAHRFKDLGEENFKALVLIAFAQYLQQCPFEDHVKLVNEVTEFAKTCVADESAENCDKSLHTLFGDELCKVASLRETYGDMADCCEKQEPERNECFLSHKDDSPDLPKLKPDPNTLCDEFKADEKKFWGKYLYEIARRHPYFYAPELLYYANKYNGVFQECCQAEDKGACLLPKIET |
