# Supplementary material for: Chimeric MHC class I– and II–restricted non-self epitopes broaden antitumor T cell reactions
Source: J Exp Med. 2025 Dec 5;223(2):e20250025. doi: 10.1084/jem.20250025 (PMC12679993; doi:10.1084/jem.20250025)
Supplement: Table S3 — shows the missense mutation epitopes in Bpmel tumor. [file jem_20250025_tables3.docx]

**Table S3: Missense mutation epitopes in Bpmel tumor (Supplement for Fig. S2 E)**

| Name of the original protein | Mutated epitops |
| --- | --- |
| AZIN1 | AQITPFYTV |
| HJURP | STPQTASTL |
| CLPTM1L | YSLQHFGFS |
| DYNC1H1 | KWLAFRVPL |
| FARSB | VYYSKTPGF |
| ARAF | CGYTFHQHC |
| PDXDC1 | LQYLGLDAI |
| PRKD3 | TICQYCTRL |
| AIFM1 | RAPSHAPFL |
| INPP5B | LSVSLHSEL |
| LANCL1 | RNFAAKSPL |
| ATP9B | SIYQGGILL |
| ATP9B | GGILLYGAL |
| LPAR1 | IMLPNLLVM |
| LPAR1(2) | CVFIMLPNL |
| HEBP1 | VSFALFPNE |
| HEBP1(2) | TVPVSFALF |
| COL6A3 | TAIVNLTPA |
| COL6A3(2) | SLLLSGIAM |
| COL6A3(3) | FVVENHFTQ |
| PLPP5 | CSQPFMRVL |
| PCMTD1 | SAVEDNDDL |
| UGT1A6A | FFFNCQSLL |
| UGT1A6A(2) | MFFFNCQSL |
| UGT1A6A(3) | MFFSSCQSL |
| TECPR1 | WAITNDRKV |
| TECPR1(2) | AGVSLVWAI |
| SPSB4 | VTMRYINAL |
| BYSL | MAQRFYTLV |
| BYSL | RMAQRFYTL |
| GMEB1 | SSIEGTEDM |
| ZFP966 | KAFARSSTL |
| ERBB2 | KIFGRLAFL |
| RAP1GAP2 | LANSSDVPL |
| CELF4 | YGAYAQAPM |
| Scap | VIYVLART |
| Lpar1 | VFIMLPNL |
| Nob1 | ISSRWAIL |
| Atp9b | GILLYGAL |
| Snx21 | SSRLSATL |
| Plpp5 | CSQPFMRV |
| Ugt1a6a | FFFNCQSL |
| Cdk13 | LSPRQPPL |
| Hebp1 | VSFALFPN |
| OVAI | SIINFEKL |

Red text indicates mutated amino acids.
