# Supplementary material for: Chimeric MHC class I– and II–restricted non-self epitopes broaden antitumor T cell reactions
Source: J Exp Med. 2025 Dec 5;223(2):e20250025. doi: 10.1084/jem.20250025 (PMC12679993; doi:10.1084/jem.20250025)
Supplement: Table S4 — shows the MHC II–restricted candidate epitope peptide of Notch2MUT. [file jem_20250025_tables4.docx]

**Table S4: MHC class II-restricted candidate epitope peptide of Notch2^MUT^**

| Peptide name | Sequence |
| --- | --- |
| NotMHCII1 | SPTLVRMEAPAPTETE |
| NotMHCII2 | RPASTEAPVVRLATSL |
| NotMHCII3 | TDANVLRASQASTVTA |
| NotMHCII4 | DANVLRASQASTVTAL |
| NotMHCII5 | SSPANALQASQGRSVK |
| NotMHCII6 | SAGTPTSAPAKSASQG |
| NotMHCII7 | HPRPASTEAPVVRLAT |
| NotMHCII8 | VPVTCSAGTPTSAPAK |
| NotMHCII9 | AGTPTSAPAKSASQGS |
| NotMHCII10 | SPANALQASQGRSVKL |
| NotMHCII11 | VSIPTIAAVPLSGLGS |
| NotMHCII12 | PRPASTEAPVVRLATS |
| NotMHCII13 | PTSAPAKSASQGSSVS |
| NotMHCII14 | APVSGISTTAPTTSVR |
