# Supplementary material for: Chimeric MHC class I– and II–restricted non-self epitopes broaden antitumor T cell reactions
Source: J Exp Med. 2025 Dec 5;223(2):e20250025. doi: 10.1084/jem.20250025 (PMC12679993; doi:10.1084/jem.20250025)
Supplement: Table S5 — shows the frameshift-derived functional peptide of Bpmel. [file jem_20250025_tables5.docx]

**Table S5: Frameshift-drived functional peptide of Bpmel**

| Gene_Name | Sequence after mutation | H2Kb binder | H2Db binder | I-Ab binder |
| --- | --- | --- | --- | --- |
| Hjurp | TWDQQQQRKQPVL* | 0 | 0 |  |
| Disp1 | VWDQAKHMPPVRTA* | 0 | 0 |  |
| Sp4 | FFQQ* | 0 | 0 |  |
| Zfp148 | N* | 0 | 0 |  |
| Dopey2 | RLAEPLDRPSWGPADSCGCHRVEQKAGTAPQQDQDGPRGKHVPAHPCRPGLCSQHPADRLSAAAGEGGGEEASADQRG* | 0 | 0 |  |
| Usp49 | AGAAAAGGGAGAQEGGGAAAAPRGQAAAAGGAGQCAAAEECAPAPARSRARGRAPCHPRYLPALVRSCPQSAPPAGGGPRCHRPAQPGEYLLHELHPPSAQPPPEVPGMFPEPRPFHLRAPVSPSNQREGAALW* | 1 | 1 | 1 |
| 6330408A02Rik | QRVLMATECCWRVNPGIGQGWRKRRGGAQRPATVEGLIERVRKGGDCT* | 0 | 0 |  |
| Phlpp2 | CPHSAGTRGSVCRPSRPGRGSEGADEAAPGGQA* | 0 | 0 |  |
